# Computer-Facilitated Screening and Brief Intervention for Alcohol Use Risk in Adolescent Patients of Pediatric Primary Care Offices: Protocol for a Cluster Randomized Controlled Trial

**DOI:** 10.2196/55039

**Published:** 2024-03-26

**Authors:** Lydia A Shrier, Madison M O'Connell, Alessandra Torres, Laura P Shone, Alexander G Fiks, Julia A Plumb, Jessica L Maturo, Nicholas H McCaskill, Donna Harris, Pamela J Burke, Thatcher Felt, Marie Lynd Murphy, Lon Sherritt, Sion Kim Harris

**Affiliations:** 1 Division of Adolescent/Young Adult Medicine Boston Children’s Hospital Boston, MA United States; 2 Department of Pediatrics Harvard Medical School Boston, MA United States; 3 Primary Care Research American Academy of Pediatrics Itasca, IL United States; 4 Shone Sciences, DBA Lowville, NY United States; 5 Department of Pediatrics Children’s Hospital of Philadelphia Philadelphia, PA United States; 6 School of Nursing Bouvé College of Health Sciences Northeastern University Boston, MA United States; 7 Yakima Valley Farm Workers Clinic Grandview, WA United States; 8 Elmwood Pediatric Group Rochester, NY United States; 9 Cornerstone Systems Northwest Lynden, WA United States

**Keywords:** alcohol, substance use, adolescent, primary care, prevention, intervention, screening, brief intervention, computer-facilitated screening and brief intervention, cSBI, mobile phone

## Abstract

**Background:**

Alcohol and other substance use disorders usually begin with substance use in adolescence. Pediatric primary care offices, where most adolescents receive health care, are a promising venue for early identification of substance use and for brief intervention to prevent associated problems and the development of substance use disorder.

**Objective:**

This study tests the effects of a computer-facilitated screening and brief intervention (cSBI) system (the *CRAFFT [Car, Relax, Alone, Forget, Family/Friends, Trouble] Interactive System* [CRAFFT-IS]) on heavy episodic drinking, riding with a driver who is substance impaired, or driving while substance impaired among adolescents aged 14 to 17 years presenting for a well visit at pediatric primary care practices.

**Methods:**

We are conducting a cluster randomized controlled trial of the CRAFFT-IS versus usual care and recruiting up to 40 primary care clinicians at up to 20 pediatric primary care practices within the American Academy of Pediatrics (AAP) Pediatric Research in Office Settings network. Clinicians are randomized 1:1 within each practice to implement the CRAFFT-IS or usual care with a target sample size of 1300 adolescent patients aged 14 to 17 years. At study start, intervention clinicians complete web-based modules, trainer-led live sessions, and mock sessions to establish baseline competency with intervention counseling. Adolescents receive mailed recruitment materials that invite adolescents to complete an eligibility survey. Eligible and interested adolescents provide informed assent (parental permission requirement has been waived). Before their visit, enrolled adolescents seeing intervention clinicians complete a self-administered web-based CRAFFT screening questionnaire and view brief psychoeducational content illustrating substance use–associated health risks. During the visit, intervention clinicians access a computerized summary of the patient’s screening results and a tailored counseling script to deliver a motivational interviewing–based brief intervention. All participants complete previsit, postvisit, and 12-month follow-up study assessments. Primary outcomes include past 90-day heavy episodic drinking and riding with a driver who is substance impaired at 3-, 6-, 9-, and 12-month follow-ups. Multiple logistic regression modeling with generalized estimating equations and mixed effects modeling will be used in outcomes analyses. Exploratory aims include examining other substance use outcomes (eg, cannabis and nicotine vaping), potential mediators of intervention effect (eg, self-efficacy not to drink), and effect moderation by baseline risk level and sociodemographic characteristics.

**Results:**

The AAP Institutional Review Board approved this study. The first practice and clinicians were enrolled in August 2022; as of July 2023, a total of 6 practices (23 clinicians) had enrolled. Recruitment is expected to continue until late 2024 or early 2025. Data collection will be completed in 2025 or 2026.

**Conclusions:**

Findings from this study will inform the promotion of high-quality screening and brief intervention efforts in pediatric primary care with the aim of reducing alcohol-related morbidity and mortality during adolescence and beyond.

**Trial Registration:**

ClinicalTrials.gov NCT04450966; https://www.clinicaltrials.gov/study/NCT04450966

**International Registered Report Identifier (IRRID):**

DERR1-10.2196/55039

## Introduction

### Background and Rationale

Alcohol and other drugs are a major contributor to morbidity and mortality each year [1]. Most substance use disorders (SUDs) have a pediatric origin, and substance use remains among the most prevalent of adolescent risk behaviors [2-4]. In addition, motor vehicle crashes remain a leading cause of death among adolescents, and alcohol- or drug-impaired driving and riding with a driver who is substance impaired play a major role [5].

Pediatric primary care offices, where the majority of adolescents receive health care [6], are a promising venue for early identification of problematic substance use among adolescents and for brief intervention to prevent further problems. A substantial and increasing proportion of adolescents see a primary care clinician yearly [7] and have trusting, longitudinal relationships with their clinicians [8]. Primary care visits are opportunities for private conversations that support adolescents’ autonomy and confidentiality while placing topics such as substance use in a nonpejorative health risk context.

The American Academy of Pediatrics (AAP) recommends alcohol, tobacco, and other drug use annual screening as part of routine care starting at age 11 years [9,10]. However, a national survey found that only approximately half of adolescents who had a past-year visit with a physician reported being asked about alcohol use [11,12]. Even when substance use screenings are performed, they can be of low quality if clinicians do not use a structured, validated screening tool or if they ask questions while caregivers are present [13,14]. Screenings also seldom include questions about substance-impaired driving or riding with a driver who is substance impaired [15]. Pediatric primary care clinicians also face barriers to brief intervention based on screen responses, including insufficient time during the appointment, a lack of familiarity with validated screening tools, and a lack of experience with managing care for patients who screen positive [16-18].

To overcome these barriers, digital technology can be applied to support consistent, effective screening and tailored intervention for adolescent substance use using evidence-based tools and approaches [19,20]. Clinicians may prefer to use technological tools such as tablet computers for previsit screening and electronic medical record–embedded decision support to enable brief intervention delivery [21]. Accordingly, we designed the CRAFFT (Car, Relax, Alone, Forget, Family/Friends, Trouble) Interactive System (CRAFFT-IS) to increase the frequency and quality of screenings in pediatric primary care, support a personalized response to results, and efficiently address alcohol and drug use and associated riding risks [22]. The CRAFFT-IS comprises a computer self-administered screening for patients (using the well-validated and widely used CRAFFT screening tool [23-25]), followed by psychoeducational content on the health risks of substance use, which adolescents complete before the clinician encounter. Clinicians access a computerized dashboard to view their patient’s screening results and prompts for brief motivational interviewing (MI)–based counseling tailored to their patient’s screening responses (clinician report).

CRAFFT-IS development was informed by the information-motivation-behavioral skills model [26], the health belief model [27,28], and social cognitive theory [29,30]. These models posit that individuals’ health risk behaviors are predicted by their *attention* to the behavior, *knowledge* about its health impacts, perceived *severity* of the harms that could result, perceived *benefits* of avoiding the behavior, and perceived *self-efficacy* to avoid the behavior. The CRAFFT-IS uses an MI-based approach to alert patients’ attention to the topic of alcohol use within a health context, enhance their awareness and perceived severity of the potential health harms of alcohol use, and boost their motivation and self-efficacy to avoid use.

We tested an initial version of the CRAFFT-IS among adolescent primary care patients at 9 practices in 3 New England states (Connecticut, Massachusetts, and Vermont) using a quasi-experimental design in which each office served as its own historical control (usual care [UC] phase followed by intervention phase; details reported elsewhere) [31-34]. Compared to UC patients, intervention patients reported double the rate of receiving clinician advice about alcohol and, among those with prior drinking at baseline, had one-third lower drinking risk in the 3-month follow-up [31]. We also found a 22% lower risk for heavy episodic drinking (HED) among those with prior HED at baseline and a 30% lower risk for riding with a driver who is substance impaired [33].

We then enhanced the clinician advice to include brief MI strategies (eg, eliciting a patient’s own reasons for avoiding use) while keeping the counseling brief enough for delivery by busy primary care clinicians. We pilot-tested this enhanced system compared to UC among adolescent patients seen for well visits at 5 Boston-area pediatric practices in a patient-randomized controlled trial. Intervention patients reported a 21% higher rate of receiving counseling about alcohol. Those reporting prior drinking at baseline (n=192) had a 34% lower risk for reporting HED during the 12-month follow-up [22]. Furthermore, those with riding risk at baseline (n=99) had a 42% lower risk of reporting riding with a driver who is substance impaired at the 12-month follow-up [35]. The CRAFFT-IS was also highly acceptable to the clinicians in the study, with 90% of the 49 clinicians agreeing that the system was useful, and 82% reporting that it increased their confidence in providing brief counseling [36].

To evaluate the replicability of these findings in a larger, more geographically diverse sample, we are now conducting a multisite effectiveness trial of the CRAFFT-IS among adolescent patients aged 14 to 17 years presenting for a well visit at practices within the national AAP Pediatric Research in Office Settings (PROS) network. This trial uses a clinician-randomized design, plans an adequately powered sample, and tests the intervention more broadly beyond New England. The CRAFFT-IS being tested in this study includes an updated version of the CRAFFT screen that includes nicotine vaping, now a leading form of substance use among adolescents [37]. It also includes updated psychoeducation material to reflect more current scientific knowledge and a revised brief counseling protocol to enhance alignment with MI-based counseling approaches.

### Objectives

The primary objective of the Adolescent Substance Use Prevention and Intervention Research (ASPIRE) study is to test the effect of the CRAFFT-IS on past 90-day HED over 12 months’ follow-up among adolescents who report past 12-month drinking at baseline. Our secondary objective is to test the effect of the CRAFFT-IS on riding with a driver who is substance impaired or driving while substance impaired (*riding or driving risk*) over 12 months’ follow-up. Additional exploratory study objectives include evaluating (1) hypothesized effect mediators (eg, perceived risk of harm and refusal self-efficacy) and moderators (eg, baseline risk level and gender), (2) efficacy for reducing the use of other commonly used substances among adolescents (ie, nicotine and cannabis), and (3) efficacy for reducing the negative consequences of substance use.

## Methods

### Study Team

This study is being performed in the national AAP PROS network of pediatric primary care practices.The AAP PROS network consists of pediatrician members whose guiding mission is to improve the health of children and enhance primary care practice by conducting and fostering national collaborative practice-based research. Boston Children’s Hospital (BCH) investigators are conducting the study in collaboration with the AAP PROS team. BCH conducts patient recruitment; participant retention; and data collection, management, and analysis activities. BCH also provides training and fidelity monitoring for clinicians delivering the intervention and convenes the data safety and monitoring board (DSMB). The AAP PROS team leads practice and clinician recruitment and data collection activities and hosts 2 PROS-member pediatrician advisors who provide input on study decisions from the perspective of community-based pediatric primary care clinicians.

### Ethical Considerations

The AAP Institutional Review Board (IRB) is the single IRB for the study. The AAP IRB initially approved this study on May 27, 2022, after full board review (#22 HA 01). Approval included a waiver of parental permission and a partial waiver of Health Insurance Portability and Accountability Act (HIPAA) authorization. We sought a partial waiver of HIPAA authorization because patient recruitment activities involve the exchange of patient health information from practices to the BCH study team. Furthermore, we sought a waiver of parental permission due to the minimal risk nature of the study, the developmental maturity of the target sample (adolescents aged 14-17 years), and the sensitivity of the substance-related topic. Our previous study on consent in adolescent substance use research showed that requiring parental permission resulted in a substantially higher study refusal rate (60% vs 20%) and a sample biased toward lower levels of substance use and substance-related problems [38]. Importantly, recruitment and assent materials state the voluntary nature of the study, emphasize that eligible adolescents *do not* have to have used alcohol or other substances to join the study, and encourage—but do not require—adolescents to discuss the study with their caregivers. The combination of these conditions supports parental involvement, preserves the privacy and confidentiality of adolescents who *do* use alcohol or other substances, and acknowledges adolescent autonomy to make an informed decision about participating.

The protocol version described in this paper was approved on June 21, 2023. Should there be modifications to the approved protocol or study materials, we will submit an amendment to the AAP IRB, and if applicable, the relying IRB at BCH. In addition, we will report scientific changes to the National Institute on Alcohol Abuse and Alcoholism (NIAAA) as needed or in the yearly progress report, at which point we will also update the trial registry on ClinicalTrials.gov.

In this trial, patient privacy and confidentiality protection is supported by executing a data transfer agreement between participating practices and BCH, assigning patients a numeric study identifier and by storing identifiable data in secure and restricted-access network locations separate from survey data. This study operates under a National Institutes of Health certificate of confidentiality. Assent materials state that only deidentified data will be shared outside of the study team (refer to the Data Access and Dissemination subsection).

Participating clinicians and practice staff members are required to provide informed consent. AAP PROS staff will send study recruitment materials to the designated contact clinician at each potentially eligible practice. Practices that are interested in participating will have a designated lead clinician submit a practice intake survey to confirm study eligibility of the practice and its clinicians. If practices are eligible, the AAP PROS staff will obtain written informed consent from all participating clinicians (Multimedia Appendix 1) as well as designated practice staff members who will handle and exchange patient data, answer patient or family questions, and otherwise support study activities at their site. Each participating practice will be offered a US $1000 honorarium after concluding patient recruitment at their practice and be able to keep all intervention-related materials, including the tablet computers provided for CRAFFT-IS implementation. Clinicians will be offered lunch accommodations (up to US $25 per person) for attending study orientation training and debriefing focus groups. In addition, clinicians in both trial arms can receive up to 12 continuing medical education credits for completing study training activities (UC clinicians will be offered CRAFFT-IS training at study completion at their practice). Board-certified pediatricians in the intervention arm may also receive optional maintenance of Certification Part 4 (up to 25 points) for a quality improvement activity spanning at least 3 quarterly feedback cycles. To complete this certification, intervention clinicians receive their participating patients’ aggregated postvisit data reporting receipt of counseling and compare their performance with a standardized benchmark. Intervention clinicians receive a US $5 electronic merchandise gift card for attending an optional *office hour* with the trainers by videoconference every 1 to 2 months and US $10 for completing a debriefing survey about their experience of, and views about, the CRAFFT-IS at study completion.

Adolescents must provide verbal informed assent to participate (Multimedia Appendix 2). On a regular schedule (eg, every 2 weeks), each practice will send a BCH research assistant (RA) a list of age-eligible adolescent patients with upcoming well visits in the next 2 to 6 weeks. The BCH RA will then mail a study invitation coaddressed to adolescents and their caregivers, which includes an informational letter on the practice’s letterhead and a study flyer with a QR code that leads to the brief study eligibility survey. Recruitment materials emphasize that adolescents do not need to have used substances to join the study, just as those with riding risk do not need to have used a substance themselves. Within 2 weeks of an adolescent’s scheduled visit, the RA will attempt to contact the adolescent by telephone to invite them to complete the eligibility survey, which the RA, based on the adolescent’s preference, can administer verbally or send for self-completion using an electronic link by SMS text message or email. At any point, a caregiver or adolescent can opt out of being contacted further. Eligible patients will be directed to an electronic information sheet and a 5-minute video brochure developed in collaboration with youth advisors. During a telephone call before the adolescent patients’ visit, a BCH RA will summarize key information using a standard assent script. If willing to join the study and able to answer 6 questions assessing study understanding correctly, adolescents will have the option to discuss the study with a parent or guardian and may then provide their verbal assent to participate. Adolescents who turn 18 during the study period will be asked to consent as an adult on or near their 18th birthday.

In addition to obtaining study assent, we will ask adolescents whether they assent or decline to have their deidentified study data shared with the NIAAA Data Archive (NIAAA_DA_), a national data repository to which NIAAA-funded investigators conducting human subjects research are expected to submit deidentified individual-level data [39]. This data sharing is not a condition of study participation and does not require parental permission. Adolescents can receive up to US $100 in small-value merchandise electronic gift cards for completion of study surveys (US $5, US $10, or US $15 for a survey, depending on its length).

### Study Design and Randomization

The study is a cluster randomized controlled trial involving 2 arms, intervention (CRAFFT-IS) and control (UC), with prospective follow-up for 12 months. Clinician randomization occurs within practice and on a rolling basis as practices enroll into the study. Participating pediatric primary care clinicians (doctor of medicine [MD], doctor of osteopathic medicine [DO], nurse practitioner [NP], and physician assistant [PA]) are randomly assigned to 1 of the study arms in a 1:1 ratio. Randomization is performed using a computerized, adaptive biased coin minimization scheme, which minimizes imbalance on the following factors, ranked by priority: clinician type (MD or DO vs NP or PA) and years in practice (≥10 vs <10) [40,41]. Compared to permuted blocks, adaptive biased coin minimization randomization has superior efficiency for achieving balance across multiple factors in studies with small units of randomization.

Clinicians are assigned a unique numeric code for temporary blinding while being randomized. After randomization, clinician study arm assignments are unblinded and shared with the study team. Study arm assignments cannot be blinded because the study team needs to train clinicians according to group assignment.

### Study Population, Sample Size, and Statistical Power

#### Practices and Clinicians

We will recruit up to 20 practices within the AAP PROS network, with up to 40 clinicians enrolled across these practices. To be eligible to participate, practices must (1) have at least 2 interested and eligible clinicians (clinician eligibility criteria are presented later in this subsection), (2) have a high-speed wireless internet connection available to ensure connectivity of study tablet computers, and (3) have an adolescent patient population that is primarily English speaking (the CRAFFT-IS has, to date, been tested only in English). Practices must not (1) be a practice or continuity clinic where residents or medical students routinely provide adolescent care (because trainees may not be available for training, implementation, and data collection for the duration of the practice’s participation in the study) or (2) have recently participated or be participating in other initiatives to improve adolescent substance use screening and brief intervention in their practice. Eligible clinicians (1) must be a clinician (MD, DO, NP, or PA) who sees patients for well visits and (2) must see ≥6 adolescents aged 14 to 17 years per week, on average, or approximately 300 adolescents per year, for well visits.

#### Adolescents

We aim to enroll a minimum of 1300 adolescent participants. Eligible adolescents must (1) present for an annual well visit with a participating clinician in either study arm, (2) be aged 14 to 17 years at the time of their well visit, (3) report past 12-month alcohol use or past 12-month riding risk, and (4) have a smartphone and be willing to share their mobile phone number with the study team. We chose the minimum age of 14 years because there is low prevalence of prior alcohol use among younger adolescents (<5% of adolescents aged 12-13 years in our prior studies reported past 12-month drinking [Harris SK, unpublished data, 2019]). In addition, to be considered fully enrolled, all adolescents must complete the previsit survey before seeing their clinician, as well as the CRAFFT screen for those scheduled to see a clinician in the intervention arm. Adolescents must be willing and able to complete monthly surveys for 12 months after their well visit.

Adolescent patients must not be (1) in foster care, (2) in college or trade school at the time of their well visit, (3) currently receiving counseling or treatment for a substance use concern from a specialty clinician, or (4) perceived by their clinician to be inappropriate for the study (eg, due to neurodevelopmental delays or another medical priority at the time of visit).

We calculated the adolescent patient sample size by applying recruitment and retention rates, alcohol use and riding risk prevalence rates, intervention effect sizes, a within-clinician clustering design effect (0.90), and the clinician intraclass correlation coefficient for HED (0.008) seen in our pilot study [22]. We estimated a 37% past 12-month drinking prevalence rate, 28% riding risk prevalence rate among adolescents with past 12-month drinking, and a 5.8% riding risk rate among those with no past 12-month drinking. The 37% past 12-month drinking prevalence rate is similar to the 36.1% prevalence rate found on the 2018 Monitoring the Future Survey [42]. We applied slightly more conservative rates of participation (80% vs 82%) and retention (70% vs 75%) than those found in our pilot study to ensure that we achieve a sample size with sufficient power. With α set at ≤.05 and β at ≥.80, our power calculation indicated that a minimum analysis sample size of 888 participants with 12-month follow-up data would provide 86% power to detect an effect size of ≥20% in our primary outcome measure of HED. Applying the conservative estimate of 70% retention at 12 months, we need to recruit a minimum of 1268 participants. Thus, we estimate needing approximately 4200 adolescents to complete the eligibility survey, with 1600 (40% of those screened) meeting eligibility criteria, and 1300 (80% of those eligible) enrolling. To enroll this sample, we anticipate enrolling an average of 40 patients per clinician among up to 40 clinicians completing the study.

### Intervention

#### Overview

The CRAFFT-IS was developed to leverage the power of digital technology to provide a time-efficient and feasible way for busy pediatric practices to improve both the frequency and quality of adolescent substance use screening and counseling in primary care. To this end, the CRAFFT-IS consists of the following components: (1) previsit self-administered screen (CRAFFT version with extra questions that are related to nicotine vaping and tobacco use [CRAFFT 2.1+N]), immediately followed by (2) brief interactive psychoeducational content on substance use–related health risks, and (3) clinician delivery of brief MI-based counseling during the confidential portion of the well visit, guided by a computerized point-of-care decision support tool called the clinician report. In addition, as an intervention to reduce riding and driving risk, intervention clinicians provide all participating patients and their caregivers with the *Contract for Life* document [34,43]. These components are described in greater detail in the following subsections.

#### Previsit Screening

To complete the previsit self-administered substance use screening, adolescents will log in to a secure website via their smartphone (before or at visit) or a tablet computer (at visit only) and complete the CRAFFT 2.1+N, which assesses past 12-month substance use frequency, substance-related riding and driving, and signs of problematic use (eg, uses when alone) [44]. At a cut point of ≥2 *yes* answers, the CRAFFT is highly sensitive (91%) and specific (90%) for detecting SUD in adolescents [23]. The CRAFFT 2.1+N includes an item on past 12-month days of nicotine vaping and tobacco use and, if yes, an item on past 30-day use. Endorsement of past 30-day nicotine vaping or tobacco use is followed by the Hooked on Nicotine Checklist (HONC), a well-validated 10-item screen for detecting loss of autonomy over nicotine use in adolescents [45]. This study will use a modified HONC that incorporates nicotine vaping.

After completing the substance use screen, adolescents will be asked to choose up to 3 personal values from a prespecified list of 14 values (eg, creativity, family, honesty, and wealth), with the option to write in their own. The selected values are presented in the clinician report to support clinician-patient rapport building and patient-centered MI-based counseling.

#### Previsit Psychoeducation

Next, adolescents view brief interactive psychoeducational web pages on the health risks of substance use. These web pages illustrate the unique vulnerability of adolescents to the health harms of substance use through the presentation of scientific evidence and true-life stories of adolescents. We developed this content based on input from focus groups of youth who reported that this type of information was compelling [31]. For this study, the content was updated to include information about the health risks of nocotine vaping.

#### Clinician Report and Brief Counseling Protocol

The clinician report is a web-based dashboard designed to help clinicians efficiently view their adolescent patients’ responses to the substance use screening and values items and deliver brief counseling tailored to the responses. On the basis of the screening responses, counseling guidance is tailored to 1 of three substance use profiles: (1) *recent use* (use of alcohol, cannabis, or another drug in the past 3 months or use of tobacco or nicotine in the past 30 days), (2) *distant use* (use of any substance in the past 12 months but not in the past 3 months or past 30 days for nicotine vaping and tobacco), and (3) *riding risk* (no past 12-month substance use but past 12-month riding in a vehicle with a driver who was substance impaired).

The brief counseling protocol is a modified form of the brief negotiated interview, a structured MI-based counseling intervention originally developed for use in emergency departments and found to be efficacious in reducing substance use in adolescents [46]. The counseling protocol is designed for delivery in 5 to 10 minutes, depending on the severity of risk. Counseling steps are summarized in Table 1.

**Table 1:** Overview of the Adolescent Substance Use Prevention Intervention Research (ASPIRE) multisite cluster randomized controlled trial’s brief counseling protocol, which is delivered by intervention clinicians to participating adolescent patients who present for a well visit and report past 12-month alcohol use, past 12-month riding risk, or both.

Counseling protocol steps	Recent use^a^	Distant use^b^	Riding risk^c^
1. Engage and build rapport	✓	✓	✓
2. Review screening results and assess further	✓	✓	✓
3. Consider pros and cons of substance use (decisional balance)	✓		
4. Discuss health risks of substance use and riding risk	✓	✓	✓
5. Evaluate readiness to change substance use (readiness ruler [47]) and reasons for change	✓		
6. Elicit next steps toward behavior change and anticipate challenges	✓	✓	✓^d^
7. Wrap up by summarizing discussion and affirming autonomy and self-efficacy	✓^d^	✓^d^	✓

^a^Patient reports past 3-month substance use. Patient may or may not report past 12-month riding with a driver who was substance impaired.

^b^Patient reports past 12-month substance use but no past 3-month use. Patient may or may not report past 12-month riding with a driver who was substance impaired.

^c^Patient reports past 12-month riding with a driver who was substance impaired and no past 12-month substance use.

^d^Clinician provides patient with the *Contract for Life* [43].

*Recent use* counseling uses the MI style of communication to elicit the adolescent’s reasons for and problems with substance use. The clinician discusses the adolescent’s values, shares health information, explores the adolescent’s reasons for and readiness to change their substance use behavior using a readiness ruler [47], and guides the adolescent through planning behavior change. The counseling concludes by summarizing the discussion and affirming the adolescent’s autonomy and self-efficacy. *Distant use* counseling involves affirming and supporting the adolescent’s continued nonuse, discussing health risks associated with past substance use, and anticipating and planning for challenges with maintaining abstinence. *Riding risk* counseling also includes affirmation and support of the adolescent’s continued nonuse, discussion of health risks associated with riding with a driver who is substance impaired, and counseling to identify and plan safe transportation at all times. Both the *distant use* and *riding risk* counseling protocols are guided in a structured step-by-step manner similar to *recent use* counseling.

Near the end of the brief tailored counseling, intervention clinicians provide *all* participating patients with a paper or electronic version of the *Contract for Life*. Developed by Students Against Destructive Decisions, the *Contract for Life* asks youth and their caregivers to develop and commit to a plan to ensure that adolescents always have a safe ride home with a sober driver [43].

#### Intervention Training and Fidelity Monitoring

Two clinicians with expertise in MI train intervention clinicians to deliver the intervention counseling. Training was initially structured as 5 self-study modules on a web-based learning platform and 5 live sessions with expert trainers (approximately 7 total training hours). As scheduling the 5 live sessions was found to be challenging with the first practice, the study team restructured the training to include longer self-paced asynchronous modules to reduce scheduling challenges without changing the total number of training hours. The updated training schedule consists of 5 self-study modules with recorded lectures, 2 live counseling practice sessions, and 1 live technical training session to orient clinicians to the clinician report and tablet computers.

The self-study modules include video-recorded presentations on MI-based counseling principles, the counseling steps for each of the 3 risk categories, video-recorded mock counseling demonstrations with accompanying annotated transcripts, and self-assessment questions. In the 2 live sessions, the expert trainers guide clinicians through case-based practice of the counseling steps and provide feedback.

To establish baseline competency, each clinician completes the counseling steps with a standardized patient for each of the 3 risk categories in a single recorded videoconference session. The trainers and 2 research staff members review these recordings using a standardized rating form. The trainers then meet with each clinician for individualized feedback and coaching. For fidelity monitoring, clinicians complete mock counseling sessions and receive feedback quarterly. In addition, the intervention trainers host optional live videoconference sessions (*office hours*) every 1 to 2 months for extra coaching.

#### Intervention Modification or Discontinuation

No concomitant care and interventions are prohibited during the trial beyond those defined in the practice inclusion criteria. We will withdraw a participant from the study if there is evidence to suggest that continuing in the study may be inappropriate (ie, there are intervening medical, mental health, or social circumstances that preclude or disrupt participation). Data collected from withdrawn participants will still be used in the analysis.

### Outcomes and Measures

#### Study Activities and Assessment Timeline

**Figure 1:**
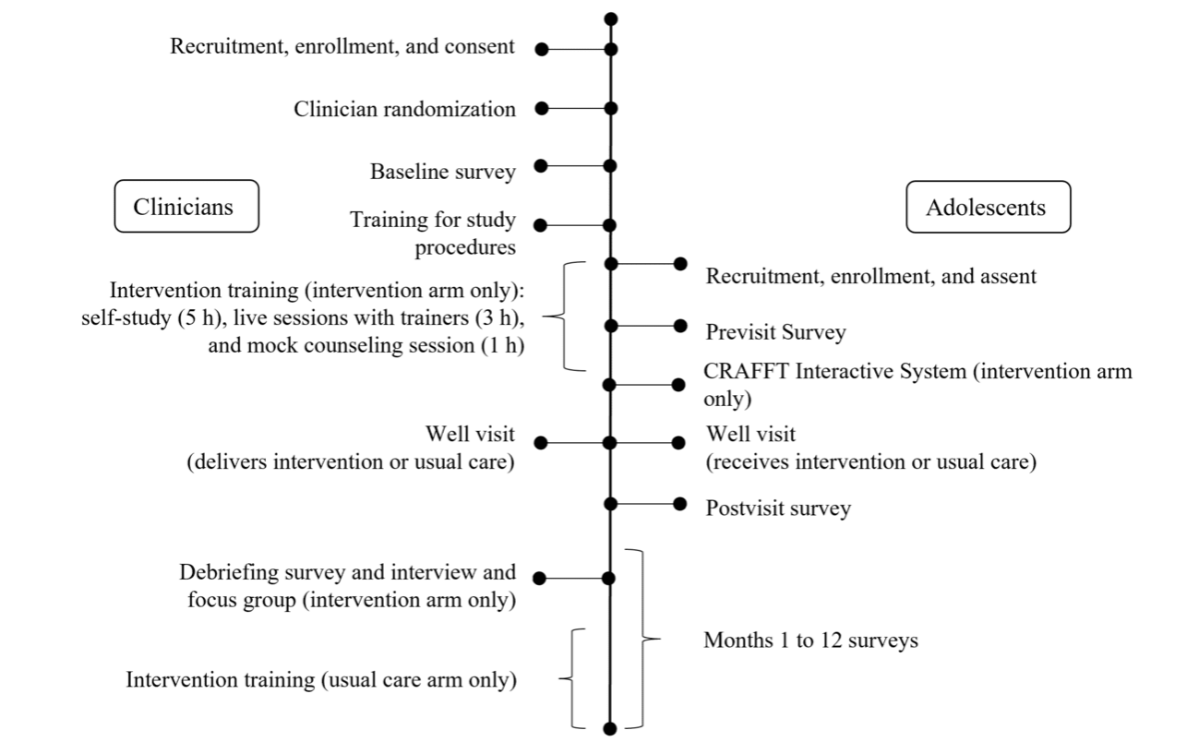
Chronological overview of clinician and adolescent milestones during the Adolescent Substance Use Prevention Intervention Research (ASPIRE) multisite cluster randomized trial. CRAFFT: Car, Relax, Alone, Forget, Family/Friends, Trouble.

Figure 1 illustrates the chronology of participating clinician and adolescent study activities.

#### Outcome Measures

A detailed listing of study outcome measures, their reference sources where applicable, and time points of data collection is presented in Table 2, and thus they are only briefly described here. These measures are also reported in the ClinicalTrials.gov registry.

Our primary outcome measure is past 90-day HED, as reported by adolescents during the 12-month follow-up period, among adolescents who report past 12-month drinking at baseline. HED is defined using the age- and sex-specific cut points recommended by the NIAAA: ≥3 drinks on a single occasion for youth assigned female at birth who are aged 14 to 17 years, ≥4 drinks for youth assigned male at birth who are aged 14 to 15 years, and ≥5 drinks for youth assigned male at birth who are aged 16 to 17 years [68]. Our secondary outcome measure is any past 90-day riding with a driver who was substance impaired (who used alcohol, cannabis, or another drug) or driving while substance impaired. The exploratory outcomes of interest include past 90-day days of use of other commonly used substances among adolescents (ie, nicotine and cannabis) and experience of negative consequences associated with alcohol use (eg, missing school or work and getting into trouble at school, home, or work). In addition, we will explore the following intermediate outcome measures as hypothesized effect mediators: (1) report of readiness to reduce or stop alcohol use using a readiness ruler [47], (2) perceived risk of harm from use, and (3) self-efficacy to refuse alcohol.

**Table 2:** Adolescent participant study assessment measures by data collection time point across 12 months in the Adolescent Substance Use Prevention Intervention Research (ASPIRE) multisite cluster randomized controlled trial.

Measure					Data collection time point																								
					Eligibility survey			Previsit survey			CRAFFT-IS^a^			Postvisit survey			Monthly^b^			3 mo			6 mo			9 mo			12 mo
**Primary outcomes**																													
	**Heavy episodic drinking^c^**																												
		TLFB^d,e^ [48,49]					✓									✓			✓			✓			✓			✓	
		AUDIT-C^f,e^ [50]					✓									✓			✓			✓			✓			✓	
	Substance use^g^ (CRAFFT^h^ [24,44] on eligibility and TLFB all other times)			✓			✓									✓			✓			✓			✓			✓	
	**Negative consequences**																												
		Negative consequences of alcohol and cannabis use scales^e^ [51]					✓												✓			✓			✓			✓	
		PEI^i^ Personal Consequences Scale^e^ [52,53]					✓												✓			✓			✓			✓	
		NCANDA^j^ study^e^ [54]					✓												✓			✓			✓			✓	
**Secondary outcome**																													
	**Riding and driving risk frequency**																												
		National College Alcohol Study^e^ [55]					✓												✓			✓			✓			✓	
		Young Adult Driving Questionnaire^e^ [56]					✓												✓			✓			✓			✓	
		Adapted from previous cSBI^k^ studies [22,54]					✓												✓			✓			✓			✓	
**Other measures**																													
	**Hypothesized mediators**																												
		Readiness to reduce or stop use (readiness ruler [47] used in previous cSBI study [22])					✓						✓																
		Perceived risk of harm (National Monitoring the Future Survey^e^ [37])					✓						✓						✓			✓			✓			✓	
		Refusal self-efficacy (DRSEQ-SRA^l,e^ [57])					✓						✓						✓			✓			✓			✓	
		Receipt of health services for substance use (adapted from previous cSBI study [22])																	✓			✓			✓			✓	
	**Control variables**																												
		**Substance use severity profile**																											
			CRAFFT	✓					✓																				
			TLFB			✓									✓			✓			✓			✓			✓		
			ASSIST^m,e^ [58]			✓												✓			✓			✓			✓		
		Peer substance use (PEI)					✓												✓			✓			✓			✓	
		Family substance use (PEI)					✓															✓						✓	
		**Patient-clinician relationship**																											
			Number of previous visits with clinician									✓																	
			Youth Connectedness to Provider Scale [8,31]									✓																	
	**Process measures**																												
		Intervention duration (new)								✓																			
		Visit format (new)											✓																
		Protocol adherence and visit quality (adapted from previous cSBI studies [8,31])											✓																
		*Contract for Life* [43] discussion with caregivers (adapted from previous cSBI study [35])																	✓										
	**Validation of modified HONC^n^**																												
		**Nicotine use disorder risk**																											
			HONC^e^ [45,59]						✓																				
			PROMIS-E^o,e^ [60]			✓																							
	**Other exploratory variables**																												
		**Quality of life and overall health**																											
			CHU9D^p^ [61]			✓												✓			✓			✓			✓		
			PATH^q^ Study [62]			✓												✓			✓			✓			✓		
			YRBS^r,e^ [63]			✓												✓			✓			✓			✓		
		Sexual and gender minority status (YRBS and others [64-66])		✓															✓			✓			✓			✓	
		**Social determinants of health**																											
			MSPSS^s^ [67]			✓												✓			✓			✓			✓		
			YRBS			✓												✓			✓			✓			✓		

^a^CRAFFT-IS: CRAFFT (Car, Relax, Alone, Forget, Family/Friends, Trouble) Interactive System (collected before the well visit among intervention arm patients only).

^b^Monthly surveys 1, 2, 4, 5, 7, 8, 10, and 11 are brief in length; monthly surveys 3, 6, 9, and 12 are extended in length.

^c^Defined by the National Institute on Alcohol Abuse and Alcoholism Alcohol Screening and Brief Intervention for Youth Guide [68].

^d^TLFB: timeline followback.

^e^Modified or adapted for purposes of this study.

^f^AUDIT-C: Alcohol Use Disorders Identification Test–Concise.

^g^Includes alcohol, nicotine, tobacco, marijuana, inhalants, misused prescription medication, and other drugs (synthetic marijuana, stimulants, opioids, and hallucinogens).

^h^CRAFFT: Car, Relax, Alone, Forget, Family/Friends, Trouble.

^i^PEI: Personal Experience Inventory.

^j^NCANDA: National Consortium on Alcohol and Neurodevelopment in Adolescence.

^k^cSBI: computer-facilitated screening and brief intervention.

^l^DRSEQ-SRA: Drinking Refusal Self-Efficacy Questionnaire–Shortened Revised Adolescent version.

^m^ASSIST: Alcohol, Smoking and Substance Involvement Screening Test.

^n^HONC: Hooked on Nicotine Checklist.

^o^PROMIS-E: Patient-Reported Outcomes Measurement Information System Nicotine Dependence Item Bank for Electronic Cigarettes.

^p^CHU9D: Child Health Utility–9 Dimensions.

^q^PATH: Population Assessment of Tobacco and Health.

^r^YRBS: Youth Risk Behavior Survey.

^s^MSPSS: Multidimensional Scale of Perceived Social Support.

#### Other Measures

We will explore potential intervention effect moderation by age group, sexual and gender identity and social determinants of health that enhance substance use risk for sexual and gender minority youth, baseline severity of substance use involvement, peer and family substance use, and patient-clinician relationship (number of prior visits the patient had with this clinician and perceived connectedness to their clinician).

We will also examine intervention implementation measures, including the time required to deliver the various CRAFFT-IS components and patient-reported receipt and quality of substance use counseling during their visit. Time information will be collected through computerized capture of patient clickstream times and clinician counseling start and stop times, as well as clinician report of average substance use–related counseling time and overall visit length on the clinician debriefing questionnaire completed at the end of patient recruitment at a practice. Adolescents complete an immediate postvisit questionnaire that includes items on the health topics discussed during the visit, ratings of counseling quality and the degree to which it reflected an MI-based counseling style, and whether they received the *Contract for Life*.

To assess the validity of the modified HONC for identifying risk for nicotine use disorder in adolescents, we will compare it to the 4-item Patient-Reported Outcomes Measurement Information System Nicotine Dependence Item Bank for Electronic Cigarettes (PROMIS-E) [60] administered in the previsit assessment. While the CRAFFT screening items are well validated [24], the modified HONC, which now includes nicotine vaping and is included in the CRAFFT-IS previsit screening for adolescents seeing intervention clinicians, needs validation.

#### Data Collection

##### Clinicians

Enrolled clinicians are assigned a unique numeric identifier for use in data collection throughout the study. A baseline survey assesses previous experience and training and UC practices around screening, brief intervention, and referral to treatment (10 min), as well as training on study procedures and human subjects protections (up to 90 min; all clinicians). Clinicians assigned to the intervention arm additionally complete brief (5 min each) postsession evaluation surveys after each live training session and a final summative evaluation after all training components have concluded. Clinician surveys and evaluations are completed on paper or by email or SMS text message link to an online survey per clinician preference. After adolescent recruitment and intervention delivery have concluded at a practice, intervention clinicians complete a 10-minute debriefing survey assessing experience using the CRAFFT-IS and seeking suggestions for improving the system, as well as participate in a 30- to 60-minute interview or focus group (along with participating staff) to offer feedback on their study experience.

##### Adolescents

All adolescent participants are assigned a unique numeric identifier at the time of recruitment for use in data collection, storage, and linkage throughout their study participation. Once assented, participants indicate their contact preferences. Adolescents who agree to have their data shared with the NIAAA_DA_ are assigned a Global Unique Identifier using their first, middle, and last names; sex assigned at birth; and date and city of birth. Global Unique Identifiers are numeric codes that allow participant data to be submitted to the NIAAA_DA_ without any personally identifiable information. All participants self-administer the confidential electronic previsit survey (approximately 20 min; refer to Table 2 for details on all surveys), and participants seeing an intervention clinician complete the screening and psychoeducational components of the CRAFFT-IS (5 min) before seeing their clinician. After their visit, all participants complete the immediate postvisit survey (approximately 10 min), and then, through the 12-month follow-up period, brief monthly surveys about their past 30-day substance use and longer surveys at 3, 6, 9, and 12 months (approximately 15 min each). Participants may choose to skip questions or entire surveys and still remain in the study.

All surveys can be completed electronically on smartphones and desktop or tablet computers through a link sent by SMS text message or email to participants’ personal mobile phone numbers or email addresses, as preferred. Electronic surveys are protected by a password set by the adolescent. Adolescents can also complete a survey by mobile phone with a study RA. If the adolescent does not complete a survey after electronic reminders, a study RA will call the adolescent to encourage survey completion.

#### Data Management

Study data, including survey responses and internal study tracking data, will be collected and stored in Research Electronic Data Capture (REDCap), a HIPAA-compliant web-based data management and survey distribution tool hosted at BCH [69,70]. Data transmission among BCH, AAP PROS staff, and practices will occur securely via password-protected email or fax. Paper-based data (eg, faxes and verbally administered surveys) will be stored securely for 7 years and identifiable electronic data for 3 years. Deidentified data will be kept indefinitely.

#### Data Analysis Plan

Analysts will be blinded to treatment arm. We will evaluate randomization success and level of attrition bias by comparing baseline characteristics of adolescents by treatment arm and by those retained versus those lost to follow-up (ie, adolescent participants who stop completing study surveys during the 12 months after their well visit). Baseline variables meeting a *P* value of <.20 in randomized group comparisons will be entered as control variables in multivariable modeling of the intervention effect to yield adjusted estimates of effect. We will compare rates of counseling receipt and patient ratings of their visit between groups. We will also generate variables related to intervention *dose* (eg, receipt of clinician counseling and receipt of *Contract for Life*) for use in sensitivity analyses.

To assess intervention effects on our primary and secondary outcome measures (past 90-day HED and any past 90-day riding or driving risk) at each of the 3-, 6-, 9-, and 12-month follow-up time points, we will use multiple logistic regression modeling with generalized estimating equations to compute adjusted relative risk ratios (CRAFFT-IS vs UC). Generalized estimating equations account for clustering of participants within practice and clinician. Furthermore, we will explore intervention effects on the number of days of drinking and of HED and on the number of times participants experienced alcohol-related negative consequences from baseline to each follow-up time point. As these variables are likely to have distributions that are highly skewed and overdispersed, we will specify a negative binomial distribution and log link in these regression models. We will also conduct longitudinal data analysis using mixed effects modeling to compare group outcomes trajectories through the entire 12-month follow-up period. We plan to explore intervention effect mediation by applying the product of coefficients test for mediation [71,72] and potential effect moderation by future-determined variables by testing the significance of interaction terms in regression models.

All intervention effect analyses will use intent-to-treat groups. We will transform continuous variables with skewed data and collapse categories as needed to preserve adequate cell sizes. To handle missing data, we will use multiple imputation based on regression modeling and compare the results of analyses using the imputed data set versus the original data set [73].

### Safety and Monitoring

We define the following as nonemergency safety risks: drinking twice the NIAAA-defined HED amount for the adolescent’s age and sex [68], daily use of alcohol or cannabis, use of other drugs on >6 days during the past 3 months, driving after use of substances >1 time in the past 3 months, and riding with a driver who is substance impaired >1 time in the past 3 months. When an adolescent’s survey responses indicate any of these nonemergency risks, a BCH RA will notify the patient’s clinician within 1 business day. If an adolescent spontaneously discloses an emergency safety risk (eg, abuse, suicide risk, or intentions to harm others), a BCH RA will immediately notify their clinician or appropriate contact person according to site-specific preferences.

This study has an independent DSMB that consists of 3 members: a child psychiatrist with expertise in adolescent SUD screening and treatment research (chair), a biostatistician with experience in clinical trial research, and an adolescent medicine primary care clinician. The DSMB will (1) review study procedures and progress, including recruitment, retention, and dropout; and (2) assess adverse events and unanticipated problems, if any, and their relationship to study participation. The full DSMB will meet annually to review overall study progress and will also convene as needed to review and address any adverse events or other study-related problems, should they arise. The DSMB is responsible for recommending whether to stop the study; no interim analyses are planned.

Given that this is a minimal risk study, we do not anticipate that participants will experience harm from study participation. However, we have planned for situations in which participants spontaneously report harms. Harms may consist of nonserious adverse events (eg, participant complaints or upset related to study activities and inadvertent disclosure of confidential information) or serious adverse events (eg, motor vehicle crashes and hospital visits). If we learn of an adverse event, we will consult with the DSMB chair and notify the AAP IRB and the patient’s practice. The DSMB will determine whether a serious adverse event is related to study participation and recommend the appropriate response, including possible study withdrawal. Study data are protected by a certificate of confidentiality from the National Institutes of Health.

## Results

### Recruitment and Enrollment

The first practice enrolled in August 2022. As of July 2023, a total of 6 practices (23 clinicians) had enrolled in the study.

Adolescent recruitment began in December 2022 and is expected to continue until late 2024 or early 2025. Data collection is expected to conclude in 2025 or 2026, and data analysis will follow.

### Data Access and Dissemination

The final trial deidentified data set will be provided to the study’s biostatistician, the AAP PROS team, and any other interested investigators upon request. We will report results on ClinicalTrials.gov, and select deidentified data will be available to researchers worldwide via the NIAAA_DA_.

In addition to this paper, we aim to publish at least 2 papers that disseminate study findings: 1 focused on our primary alcohol use–related outcomes and 1 focused on other substance use outcomes. We also anticipate submitting at least 2 abstracts to present at national conferences. Members of the study team will develop and write all publications resulting from this study. Authorship will be designated according to those who offer intellectual contribution to the design or preparation of a given publication and claim responsibility for its contents.

## Discussion

The ASPIRE study aims to test the effectiveness of the CRAFFT-IS, compared to UC, in reducing alcohol use and substance use–related riding risk among adolescents aged 14 to 17 years presenting for well visits. Despite the AAP’s recommendation for universal substance use screening in primary care beginning at age 11 years, the US Preventive Services Task Force gave primary care–based adolescent alcohol screening and brief behavioral counseling an “I” rating in its most recent review, indicating insufficient evidence for or against its recommendation [74]. The ASPIRE study builds on prior research supporting the promise of CRAFFT-IS as a feasible, acceptable, and efficacious approach to increasing high-quality screening and counseling.

Our study protocol has limitations. Although our virtual recruitment and enrollment approach allows the study team to contact a larger volume of individuals over an expanded time frame (eg, in comparison to in-person recruitment restricted to clinic operating hours), practices may not have up-to-date contact information for adolescent patients. Likewise, it is not guaranteed that adolescents will see or read the mailed recruitment invitation before their visit. Our approach also reduces burden on practice staff to conduct recruitment activities, but it may underuse pre-established connections between practice staff and adolescent patients. Furthermore, the addition of the CRAFFT-IS to well visits may delay clinic flow. Further limitations to our study may emerge during the trial and will be reported in future outcomes manuscripts.

If the CRAFFT-IS is shown to be effective, we intend to scale, promote, and widely disseminate the instrument for use in pediatric practice, either as a tablet application or as a confidential add-on to existing electronic health record systems. We also envision this study informing future investigators interested in studying computer-facilitated screening and brief intervention for youth substance use, whether in primary care settings or elsewhere, with the aim of reducing alcohol-related morbidity and mortality during adolescence and beyond.

## Appendix

Multimedia Appendix 1 Clinician consent form.

Multimedia Appendix 2 Verbal assent script delivered to eligible adolescents.

Multimedia Appendix 3 Peer-review reports from the Clinical, Treatment and Health Services Research Review Subcommittee, National Institute on Alcohol Abuse and Alcoholism Initial Review Group (National Institutes of Health).

## Abbreviations

AAP: American Academy of Pediatrics
ASPIRE: Adolescent Substance Use Prevention Intervention Research
BCH: Boston Children’s Hospital
CRAFFT 2.1+N: CRAFFT version with extra questions that are related to nicotine vaping and tobacco use
CRAFFT: Car, Relax, Alone, Forget, Family/Friends, Trouble
CRAFFT-IS: CRAFFT Interactive System
cSBI: computer-facilitated screening and brief intervention
DO: Doctor of Osteopathic Medicine
DSMB: data safety and monitoring board
HED: heavy episodic drinking
HIPAA: Health Insurance Portability and Accountability Act
HONC: Hooked on Nicotine Checklist
IRB: institutional review board
MD: Doctor of Medicine
MI: motivational interviewing
NIAAA: National Institute on Alcohol Abuse and Alcoholism
NIAAADA: National Institute on Alcohol Abuse and Alcoholism Data Archive
NP: nurse practitioner
PA: physician assistant
PROMIS-E: Patient-Reported Outcomes Measurement Information System Nicotine Dependence Item Bank for Electronic Cigarettes
PROS: Pediatric Research in Office Settings
RA: research assistant
REDCap: Research Electronic Data Capture
SUD: substance use disorder
UC: usual care

## Data Availability

Data sharing is not applicable to this paper as no data sets were generated or analyzed.
