# Correction: Computer-Facilitated Screening and Brief Intervention for Alcohol Use Risk in Adolescent Patients of Pediatric Primary Care Offices: Protocol for a Cluster Randomized Controlled Trial

**DOI:** 10.2196/59383

**Published:** 2024-05-08

**Authors:** Lydia A Shrier, Madison M O'Connell, Alessandra Torres, Laura P Shone, Alexander G Fiks, Julia A Plumb, Jessica L Maturo, Nicholas H McCaskill, Donna Harris, Pamela J Burke, Thatcher Felt, Marie Lynd Murphy, Lon Sherritt, Sion Kim Harris

**Affiliations:** 1 Division of Adolescent/Young Adult Medicine Boston Children’s Hospital Boston, MA United States; 2 Department of Pediatrics Harvard Medical School Boston, MA United States; 3 Primary Care Research American Academy of Pediatrics Itasca, IL United States; 4 Shone Sciences, DBA Lowville, NY United States; 5 Department of Pediatrics Children’s Hospital of Philadelphia Philadelphia, PA United States; 6 School of Nursing Bouvé College of Health Sciences Northeastern University Boston, MA United States; 7 Yakima Valley Farm Workers Clinic Grandview, WA United States; 8 Elmwood Pediatric Group Rochester, NY United States; 9 Cornerstone Systems Northwest Lynden, WA United States

In “Computer-Facilitated Screening and Brief Intervention for Alcohol Use Risk in Adolescent Patients of Pediatric Primary Care Offices: Protocol for a Cluster Randomized Controlled Trial” (JMIR Res Protoc 2024;13:e55039) the authors noted some errors.

The following sentence:

The AAP PROS network consists of 67,000 pediatrician members whose guiding mission is to foster and conduct national collaborative practice-based research for improving child health.

Has been changed to:

The AAP PROS network consists of pediatrician members whose guiding mission is to improve the health of children and enhance primary care practice by conducting and fostering national collaborative practice-based research.

The author Laura P Shone’s affiliation:

Primary Care Research, American Academy of Pediatrics, Itasca, IL, United States

Has been corrected to include:

Shone Sciences, DBA, Lowville, NY, United States (current); Primary Care Research, American Academy of Pediatrics, Itasca, IL, United States (former)

Additionally, the following sentence was added to the “Acknowledgments” section:

Laura P Shone was affiliated with the American Academy of Pediatrics at the time of protocol development and is affiliated with Shone Sciences, DBA at the time of publication.

The correction will appear in the online version of the paper on the JMIR Publications website on May 8, 2024, together with the publication of this correction notice. Because this was made after submission to PubMed, PubMed Central, and other full-text repositories, the corrected article has also been resubmitted to those repositories.

