# Supplementary material for: Computer-Facilitated Screening and Brief Intervention for Alcohol Use Risk in Adolescent Patients of Pediatric Primary Care Offices: Protocol for a Cluster Randomized Controlled Trial
Source: JMIR Res Protoc. 2024 Mar 26;13:e55039. doi: 10.2196/55039 (PMC11005433; doi:10.2196/55039)
Supplement: Multimedia Appendix 1 [file resprot_v13i1e55039_app1.pdf]

# Clinician Consent Form

**Title:** The Adolescent Substance use Prevention Intervention Research (ASPIRE) Study
**Principal Investigator (PI):** Sion Kim Harris, PhD, CPH and Lydia Shrier, MD MPH
**Funder:** National Institute of Alcohol Abuse and Alcoholism (NIAAA) Grant #R01AA027253 and supplemental grants #3R01AA027253-03S1, #3R01AA027253-03S2

## KEY INFORMATION:

- Drs. Sion Kim Harris and Lydia Shrier from Boston Children's Hospital (BCH) and the American Academy of Pediatrics' (AAP) Pediatric Research in Office Settings (PROS) network are conducting the ASPIRE Study. The study has been approved by the AAP Institutional Review Board (IRB).
- The study's goal is to test a computerized-facilitated screening and brief intervention system, called **the CRAFFT Interactive,** that aims to reduce alcohol and other substance use among patients aged 14 through 17 years old.
- The study is recruiting about 36 clinicians at a maximum of 20 pediatric practices across the US.
- Participating clinicians will be randomized into one of two arms – Intervention or Usual Care.
    - Clinicians in the ***Intervention arm*** will receive training in brief Motivational Interviewing (MI) based counseling for substance use at the start of the study, along with periodic fidelity monitoring and coaching activities throughout the study. Participating patients of Intervention arm clinicians will complete questions in the online CRAFFT Interactive system, which their clinicians will use to screen them and provide brief counseling.
    - Clinicians in the ***Usual Care arm*** will be offered training in brief MI-based counseling for substance use at the end of the study. Participating patients of Usual Care arm clinicians will not use the online CRAFFT Interactive system.
- Each clinician will help recruit up to 45 of their own patients (adolescents aged 14-17 years old with an upcoming annual well visit scheduled). The patient recruitment period is approximately 18 months.
- Each clinician will participate in a survey at the start of the study. Intervention Clinicians will participate in a survey and focus group/interviews at the end of the study.
- Each clinician and their practice will be in the study for about 2 years.

*The rest of this consent form describes the ASPIRE Study and what you may expect if you decide to participate. You are encouraged to read this consent form carefully and ask any further questions before making your decision about whether or not to participate.*

**Purpose**

The overall goal of the ASPIRE Study is to test the effectiveness of a computerized-facilitated screening and brief counseling intervention system (the CRAFFT Interactive), compared to Usual Care, on reducing the following among patients aged 14 through 17 years old during a 12-month follow-up period:

- Heavy episodic drinking rates
- Rates of a key safety risk associated with alcohol use, i.e., riding with an impaired driver ("riding risk")

Additionally, the study will seek to improve representation of Sexual and Gender Minority (SGM) youth in the study sample to examine the effects of SGM identity and SGM-related Social Determinants of Health (SDoH) on that underlie the health disparities seen in SGM youth within intervention outcomes.

**Study Procedures for Clinicians in BOTH Arms**

**If you decide to participate in this study, you will agree to be randomized into either the Intervention arm or the Usual Care arm. In both arms, you will be asked to:**

1. Provide written, informed consent.

2. Complete and return PROS Practitioner Intake Form.
3. Complete a Study Box / Human Subjects Training. These trainings are designed to teach you about adolescent substance use, the purpose of the study, and how to properly conduct research activities ethically. After you complete the Human Subjects Training, you will be asked to complete and return an Individual Investigator Agreement (IIA) verifying you completed this required training.
4. Participate in a Baseline survey (by paper, or an online link that is sent via email or text message, based on your preference) before trainings and study activities begin; takes about 10 minutes.
5. Agree to help recruit up to 45 of your patients over an approximately 18-month recruitment period. *Note: Your patients are eligible for recruitment if they are aged 14 to 17 years old, can read and communicate in English, and have an upcoming well visit appointment scheduled. Additionally, your patients will be further screened by the BCH study team to verify other eligibility criteria such as past 12-month alcohol use or riding risk.*
    - Agree to share your eligible patients' contact information with the BCH study team.
    - Review lists of your eligible patients for study appropriateness and make exclusions based on your clinical judgement and knowledge.
6. Agree to implement a safety flag follow-up with an enrolled patient if the study team identifies and notifies you of a safety flag based on the patient's responses to their study surveys. The BCH study team will orient you to the safety flags (emergency and non-emergency) during your Study Box training prior to the start of patient recruitment. At this time, you and the BCH study team will develop a plan for how the study team can best notify your practice when a patient indicates a safety flag. The BCH study team will notify you within 1 business day via fax and/or phone, depending on your preference, when this safety flag occurs. If fax is preferred, a secure process will be requested.

**<u>Additional Study Procedures for Clinicians in the INTERVENTION Arm</u>**

**If you are randomized to the Intervention arm, you will be asked to:**

7. Participate in Intervention training sessions around the brief counseling intervention, so that you can implement this type of intervention at well visits with participating patients. Intervention training sessions will be offered within an approximate 8-week period <u>at the start of the study</u>. During these intervention training sessions, you will:
    - Receive up to 3 hours of live, applied MI Practice sessions, including training on how to deliver brief counseling tailored to a patient's screening results. The lead trainer is a primary care clinician with decades of experience in teaching and delivering MI-based counseling.
    - Review online self-study modules, pre-recorded MI examples, and homework.
    - Complete a video-taped standardized patient encounter and receive feedback before the start of patient recruitment.
    - During the first month of the study, attend at least one "office hour" hosted by the expert trainers.
    - Over the course of the study, improve your counseling skills by participating in up to 4 additional virtual mock sessions for fidelity monitoring and coaching.Optional office hours will be available.
    - Receive 12 CME credits upon completion of all the training sessions, should you desire.
8. Agree to use the online CRAFFT Interactive to guide your interactions with participating patients during their annual well visits. During a participating patient's well visit, you will:

- Use a study iPad or other computer device to securely access an online *Clinician Report Form*, which presents patient's substance use screening results and provides you with a tailored counseling guide, so you can review before or during the patient's visit.
    - In addition to Intervention training described above, you will receive technical training on how to use the Clinician Report Form and how to access it via iPad.
- Deliver the brief counseling intervention to patients at their visit.
- Agree to have your patient counseling timed when you access the Clinician Report Form.
- Provide patients with a copy of the Contract for Life, which encourages patients and their parents to develop a plan for safe transportation home at all times.
- *If indicated in the Clinician Report Form and/or per your discretion,* recommend that a patient returns for a follow-up visit within 3 weeks to 3 months of their well visit.

9. Receive quarterly feedback reports, sent from the BCH study team to each clinician by email or text (based on your preference), during the patient recruitment period.
    - Review feedback reports that list aggregated total rates of your patients' self-reports of having discussed certain health behaviors at their visit by:
        - Your rate
        - The study's overall Intervention arm rate
    - Complete an Attestation Form to receive 25 Maintenance of Certification (MOC) Part IV credits for participating in study activities, should you desire. Note: MOC Part IV credits only available for pediatricians.
10. Participate in a Debriefing Survey (by paper, or an online link that is sent via email or text message, based on your preference) after the patient recruitment period ends; takes about 10 minutes.
11. Participate in a Debriefing Key Informant Interview or Focus Group after the patient recruitment period ends; takes about 60 minutes.

***Study Procedures for Patients of Intervention Arm Clinicians:*** *Patients will verbally assent to the study with the BCH study team before their visit. Patients will then be asked to complete a Pre-Visit Survey and the online CRAFFT Interactive within 3 days before their well visit. If not completed on a personal device ahead of time, patients will have the option to complete the online CRAFFT Interactive via study iPad immediately before their visit. The online CRAFFT Interactive is used to screen for substance use and to review educational material about the health risks of substance use. After their visit, patients will be asked to complete an immediate Post-Visit survey and monthly surveys during the 12-month follow up period. Patients may complete surveys either by an online link (sent via email or text message), or by phone with a BCH research assistant.*

<u>**Additional Study Procedures for Clinicians in the USUAL CARE Arm**</u>

**If you are randomized to the Usual Care arm, you will be asked to:**

Participate in training sessions on brief MI-based counseling. Training sessions will be offered <u>at the end of the study</u>. If interested in these training sessions, you will:

- Complete an initial online self-study module (45 minutes) as an introduction to MI.
- Receive up to 3 hours of live, appliedMI practice sessions from expert trainers on brief MI-based counseling.
- Review online self-study modules, homework, and/or pre-recorded MI examples .
- Receive up to 12 CME credits upon completion of training sessions, should you desire.

***Study Procedures for Patients of Usual Care Arm Clinicians:*** *Patients will verbally assent to the study with the BCH study team before their visit. Patients will then be asked to complete a Pre-Visit Survey before their well visit. After their visit, patients will be asked to complete an immediate Post-Visit survey*

*and monthly surveys during the 12-month follow up period. Patients may complete surveys by online link (sent via email or text message), or by phone with a BCH research assistant.*

**Risks of Participation**

***For Clinicians/Staff:*** The risks of participation are minimal and include:

- Time required to participate in study procedures such as surveys/focus groups and reviewing lists of your eligible patients before the BCH study team recruits them.
- Time required to complete intervention training sessions. Live sessions will be scheduled based on your availability *(Intervention arm only).*
- Time required to deliver the counseling intervention to participating patients and complete other study activities as necessary *(Intervention arm only).*
- Loss of confidentiality. There is a minimal risk of loss of confidentiality, including potential disclosure of your name, your practice name, and your patients' Personal Health Information (PHI). We will implement numerous safeguards to avoid any potential breach of information (see below, Confidentiality section).

***For Patients:*** Risks for patients are also minimal. These risks include loss of confidentiality and feeling anxious or sad when answering some of the study survey questions, such as those about their substance use. However, patients may skip these questions at any time and can talk to their clinician about any feelings or concerns they may have.

**Benefits of Participation**

***For Clinicians:*** The benefit of participation for all clinicians includes learning about adolescent substance use in the Study Box and Human Subjects Training. Addressing these important issues may enhance relationships with patients and improve their care. Clinicians may also benefit from learning MI techniques, where they will have the opportunity to receive:

- One CME credit for completing the Study Box and Human Subjects Training.
- Eleven CME credits *(Intervention arm only)* for completing all intervention training sessions and fidelity monitoring/coaching activities at the start of the study.
- Twenty-five Maintenance of Certification (MOC) Part IV credits *(Intervention arm pediatricians only)* for engaging in the QI-related activities (i.e., reviewing the feedback reports, strategizing on ways to improve).
- Up to $5 electronic gift cards (e.g., Starbucks) for each time that you attend an optional monthly "office hours" meeting. Up to $10 electronic gift cards (e.g., Starbucks) for completing the debriefing survey. *(Intervention arm only*)
- Up to 12 CME credits *(Usual Care arm only)* for completing the intervention training sessions at the end of the study.

***For Patients***: Patients whose clinician is in the Intervention arm may learn about the latest science regarding the effects of alcohol and other substance use on health. Patients whose clinician is in the Usual Care arm will not receive any direct benefits from participating in the study.

**Additional Costs**

There will be no additional costs to you or your patients for participating in this research study.

**Practice Compensation**

For participating in the ASPIRE study, your practice will receive:

- $1,000 at the end of the patient recruitment period.
- Lunch for those who attend required study meetings that include both arms such (e.g., Study Box and Human Subjects Training). Lunch will be compensated up to $25 per person.
- Intervention-related materials and 1-3 study iPads provided to Intervention arm clinicians during the study, which your practice will keep after the study ends.

***Patient Compensation:*** Patients will receive electronic gift cards (based on patient preference) as a thank you for their participation. They will receive $15 for completing questionnaires related to their well visit, and up to $85 for completing the monthly follow-up surveys during the 12-month follow-up period (depending on the number of surveys completed), for a maximum total of $100.

**Confidentiality of Records**

***For Clinicians/Staff:*** While we make every effort to maintain confidentiality, it cannot be absolutely guaranteed. Records which identify you, your practice, and your patients and the consent forms signed by you may be inspected by members of the research team (PROS and BCH) and/or a regulatory agency. The results of this research study may be presented in aggregate form at meetings or in publications. We will ask for permission to use your practice name in acknowledgement, to recognize your participation in PROS and in this study. Your name will not appear in any such documents without your permission. Confidentiality of records include the following:

- All practice and clinician related study materials will be kept in secure locations in the care of PROS (surveys, consent forms, and other hard copy/electronic study materials) and BCH (survey data, other hard copy/electronic study materials, and audio-recordings with notes of debriefing interviews) for up to 7 years after the study ends. Video and audio-recording of standardized patient encounters that you may participate in after the intervention training sessions *(Intervention arm only)* will be stored securely at BCH and destroyed at the end of the study.
- Patient recruitment related data containing PHI (obtained via hard copy or electronically by your practice) will be kept in secure locations at BCH for up to 7 years after the study ends. All patients will be assigned a unique study identifier.
- No later than 7 years after the study ends, all data will be de-identified and stored in a permanent analytic dataset(s) with documentation at BCH and PROS.

Study records may be de-identified and used for future research or be given to another investigator for future research without additional consent. This will happen only with the Principal Investigators' permission.

Twilio: We will be using a service called Twilio to send you text messages (only if you opt in for this survey delivery method). This means Twilio will have access to your phone number. Twilio will not use your information for any other purpose than contacting you related to this study. However, because they keep your phone number in use logs, there is a small risk of disclosure. Because Twilio is a separate company from BCH, we do not have the ability to delete your contact information from these Twilio use logs.

This study is covered by a Certificate of Confidentiality from the National Institutes of Health (NIH). This means that the researcher team cannot give anyone information from this study that can be used against you or a patient in a legal case, unless you have agreed to this use. Also, information from this study cannot be told to anyone unless there is a federal, state, or local law that requires its release (such as to report child abuse or certain types of infection) or if it is used for other research, as allowed by federal rules protecting people participating in research.

Additionally, a description of this study will be available on ClinicalTrials.gov, as required by U.S. Law. This site will not include information that can identify you, your practice, or your patients. At most, the site will include a summary of the results. You can search this website at any time under the study title: *Computer-facilitated Screening and Brief Intervention (cSBI) in Pediatric Primary Care to Reduce Underage Drinking: a Large Multi-Site Randomized Trial.*

***For Patients:*** All identifiable patient-related study materials will be in secured locations in the care of BCH (survey data and assent) for up to 7 years after the study ends. Patient contact information will be stored separately from study data. Additionally, the study researchers will send de-identified study data to the NIAAA Data Archive ($NIAAA_{DA}$) at NIH, which is a large database where de-identified study data from many NIAAA studies are stored and managed. "De-identified" means that all personal information about your patients (such as name, address, birthdate, and phone number) is removed and replaced with a unique code number. Sharing de-identified study data helps researchers learn new and important things about alcohol problems more quickly than before. Every researcher (and institutions to which they belong) who requests de-identified study data must promise to keep the data safe and not try to learn the identity of any individual participants. Experts at NIH who know how to keep study data safe will review each request carefully to reduce risks to participants' privacy.

**Contact Persons for Information, Concerns, and To Withdraw**

- If you have questions or concerns related to your participation in this study, you may contact the Principal Investigators (PI), Drs. Sion Kim Harris and Lydia Shrier, at 617-355-5433 or aspirestudy@childrens.harvard.edu
- If you have questions about your rights as a participant, contact Erin Kelly, PhD at the AAP IRB at (630) 626-6075.
- If you agree to participate and then change your mind, you are free to withdraw your consent and stop participation at any time. Contact Drs. Harris or Shrier to withdraw.
- By consenting to participate in this study you do not waive any of your legal rights. If at any time you withdraw from this study, you will not suffer any penalty or lose any benefits to which you are entitled.

**Consent:** Your participation in this study is voluntary; you may decline or withdraw from participating without penalty at any time. Your signature below will indicate that you have read the above information, asked any questions you may have, and have received answers to those questions. You have received (or will receive) a copy of this form to keep for your records.

Study Participant (Print Name): ____________________________________________

Study Participant (Signature): ________________________ Date: ________________

Study Participant Cell Phone Number: ________________________________________

**Return a signed copy of this form to Ali Torres, MPH via *email* (atorres@aap.org and cc to prosops@aap.org), *fax* (847-434-8910) or *US Mail*. Please keep a signed copy for your files.**
