# Supplementary material for: Computer-Facilitated Screening and Brief Intervention for Alcohol Use Risk in Adolescent Patients of Pediatric Primary Care Offices: Protocol for a Cluster Randomized Controlled Trial
Source: JMIR Res Protoc. 2024 Mar 26;13:e55039. doi: 10.2196/55039 (PMC11005433; doi:10.2196/55039)
Supplement: Multimedia Appendix 2 [file resprot_v13i1e55039_app2.pdf]

Verbal Assent Scripts

An adolescent will either contact or be contacted by a BCH Research Assistant (RA) to verbally assent into the study.

Please note, the Verbal Assent Script that the BCH RA will use to obtain verbal assent is divided into:

- ***Shortened Assent Script** for adolescents who have read the Participant Information Sheet and/or viewed the Video Assent Brochure.*
- ***Extended Assent Script** for adolescents who have not read the Participant Information Sheet or viewed the Video Assent Brochure.*

	Shortened Assent Script	Extended Assent Script
**Introduction**	***If an Adolescent contacts BCH RA:***<br><br>Hi! Thanks so much for giving us a call.<br><br>We're so glad that you're eligible to join the ASPIRE study. My name is [RA NAME] and I use [LIST HERE] pronouns.<br><br>I would like to review information about the study with you and see whether you would like to join.<br><br>First, could I confirm with you, what is the 10-digit code you used when logging into the survey you just took?<br>…<br><br>Thanks. Just so you know, I'll be taking some notes during this call, so you might hear some typing. Let's get started!	***If an Adolescent is contacted by BCH RA:***<br><br>**Teen does not call within 24 hours of survey completion, so RA calls**<br><br>Hi! I'm [RA NAME] calling from Boston Children's Hospital, and I use [LIST HERE] pronouns.<br><br>Who am I speaking with today?<br>…<br><br>I saw that you recently filled out our online eligibility survey for the ASPIRE study. I am calling to follow-up. Do you have a few minutes to talk about the research study?<br>…<br><br>[IF YES] Great! Just so you know, I'll be taking some notes during this call, so you might hear some typing. Let's get started!
**Establish if adolescent reviewed Assent Materials**	At the end of the eligibility survey you completed, there were links to an information sheet and a video that describes the study. Were you able to read the info sheet or watch the video?	
	**[IF YES]** Did you have any questions about either of these?<br>…<br><br>Since you've already seen the video and info sheet, I'd like to briefly go over what you saw and ask if you have any questions.	**[IF NO]** No problem. You can always read it later and reach out to me with any questions that you have, even after our call.<br><br>I will go over the key information for you now. Feel free to ask me any questions that you have.

	Once I finish, I'll check in to see if everything made sense to you, and then you can decide whether you'd like to join the study. We'll be on the phone for about 15 minutes. Does that sound good to you?<br>…	Once I finish, I'll check in to see if the information made sense to you, and then you can decide whether you'd like to join the study. So, we'll be on the phone for about 20 minutes. Does that sound good to you?<br>…
**Study Description and Purpose**	In this study, we're testing a new online tool called the "CRAFFT Interactive." It is designed to help doctors talk about alcohol, tobacco, and other drugs with their teen patients at checkups.<br><br>Your doctor might be in the group where they will use the new tool with patients (we call this the CRAFFT Interactive group), or they might be in the group where they will keep doing what they normally do (we call this the Usual Care group).<br><br>Do you have any questions about the study's purpose? …	To start, I'd like to describe who is doing this study, where, and why. Researchers at the American Academy of Pediatrics and Boston Children's Hospital are doing this study. Dr. Sion Harris and Dr. Lydia Shrier from Boston Children's Hospital are in charge of it.<br><br>Your doctor's office is one of up to 10 offices across the U.S. in the study. We will recruit about 1,300 teens like you who are between 14 and 17 years old and have an upcoming checkup.<br><br>In the study, we're testing a new online tool called the "CRAFFT Interactive." It is designed to help doctors talk about alcohol, tobacco, other drugs with their teen patients at checkups.<br><br>Your doctor might be in the group where they will use the new tool with patients (we call this the CRAFFT Interactive group), or they might be in the group where they will keep doing what they normally do (we call this the Usual Care group).<br><br>Do you have any questions about the study's purpose?<br>…
**Voluntary Participation**	Joining this study is up to you. You don't have to join if you don't want to. Even if you say no, your doctor will still take care of you. If you do join, you can also change your mind and leave the study anytime.	Joining this study is up to you. You don't have to join if you don't want to. Even if you say no, your doctor will still take care of you. If you do join, you can also change your mind and leave the study anytime.
**Study Activities**	If you decide to join ASPIRE, you'll be asked to do several surveys over the next	If you decide to join ASPIRE, you'll be asked to do several surveys over the next

	12 months. You'll get paid for doing them, which I will describe later.<br><br>These surveys ask about your attitudes and behaviors around substance use, and about your friends and family. The surveys are password-protected, and your name is never on them.<br><br>The first survey is right before your checkup, and you might also be asked to complete the CRAFFT Interactive at this time. It's important do this survey, and if you're asked, the CRAFFT Interactive, to continue in the study.<br><br>You will also complete a survey right after your visit, and then monthly for the next 12 months.<br><br>We will send you the links to all of these surveys by text message or email, based on your preference. Please note, even if you choose email, there are some reasons why you might still get a text message during the study, like to get a verification code when setting up your password.<br><br>Do you have any questions about the different study activities? …	12 months. You'll get paid for doing them, which I will describe later.<br><br>These surveys ask about your attitudes and behaviors around substance use, and about your friends and family. The surveys are password-protected, and your name is never on them. We recommend doing surveys where you have Internet access and privacy.<br><br>We will send you the surveys by text message or email, based on your preference. Please note, even if you choose email, there are some reasons why you might still get a text message during the study, like to get a verification code when setting up your password.<br><br>First, within 3 days before your checkup, we will send the first survey, which is on a secure password-protected website.<br><br>After you complete this first survey, you may or may not be asked to complete the CRAFFT Interactive. If you are, it will take about 5 minutes. It asks you to answer a few more questions and view some educational pages. Your doctor will then see a summary report of your answers on the CRAFFT Interactive.<br><br>You must complete the survey and, if needed, the CRAFFT Interactive, before you arrive for your checkup. We'll send a few reminders to help you remember to complete them. But, if you do not complete these items before your visit, you will not be able to continue with the study.<br><br>If you have to cancel or reschedule your checkup, please let us know. We will check with your doctor's office about when your rescheduled appointment is. Then, we will ask you to re-do the pre-visit survey within 3 days of your new appointment.

		At your checkup, your doctor might talk with you about substance use.<br>Right after your checkup, we will send you a link to another survey about how your visit went. Then, over the next 12 months, we will send you a new survey once a month. These monthly surveys will have questions similar to the ones that you completed before your visit. Sometimes the surveys will be very short, like 3 minutes, and other times, they will take longer, around 15 minutes.<br><br>Do you have any questions about the different study activities? …
**Possible Benefits / Incentive**	In terms of benefits, you may learn more about your health and substance use.<br><br>You can also receive an electronic gift card each time you complete a survey, and you will get to choose between a few different retailers.<br><br>If you complete every survey, you will receive a total of $100 over 12 months.<br><br>You will receive an e-gift card after each survey you complete. There's more about this in the Information Sheet that you saw, and I'll make sure you get an extra copy of this.<br><br>Do you have any questions about the benefits, or the way payment works?<br>…	In terms of benefits, you may learn more about your health and substance use.<br><br>You can also receive an electronic gift card each time you complete a survey, and you will get to choose between a few different retailers.<br><br>If you complete every survey, you will receive a total of $100 over 12 months.<br><br>That $100 is broken down by survey, so that longer surveys are worth more dollars: $10 for the very first survey, $5 for shorter monthly surveys, $10 for the longer monthly surveys, and $15 for very last survey.<br><br>You will receive an e-gift card after each survey you complete. It's ok if you miss a survey, you just won't get paid that month. There's more about this in the Information Sheet that you saw, and I'll make sure you get an extra copy of this.<br><br>Do you have any questions about the benefits, or the way payment works?<br>…
**Possible Costs**	It doesn't cost you anything extra to join the study. For text messages, standard messaging costs from your phone carrier may apply.	It doesn't cost you anything extra to join the study. For text messages, standard messaging costs from your phone carrier may apply.

**Possible Risks**	There are some risks to joining the study. Some questions might make you feel uncomfortable or upset.<br><br>There is also a small chance that someone who isn't involved in the study tries to learn your answers. We will take several steps to keep your personal information safe.	There are some risks to joining the study. Some questions might make you feel uncomfortable or upset.<br><br>There is also a small chance that someone who isn't involved in the study tries to learn your answers. We will take several steps to keep your personal information safe.
**Privacy/ Confidentiality**	As I mentioned before, the activities you do in this study will be confidential. Your name will never be attached to any of the surveys you complete, and they will be password-protected with a password that you choose.<br><br>If you are asked to complete the CRAFFT Interactive, your doctor will see your answers. However, your doctor and your parents/guardians will not see any of your survey answers, unless any of your answers make us worried about your immediate safety. If that happens, we will tell your doctor about our concern.<br><br>Do you have any questions about the risks, or how we keep your survey answers private and confidential? …	As I mentioned before, the activities you do in this study will be confidential. Your name will never be attached to any of the surveys you complete. They will be password-protected with a password that you choose. The only people who will have access to your personal information like name or address will be our trained research staff, and they will store this information in password-protected files.<br><br>Also, your data are specially protected by something called a Certificate of Confidentiality that is given by the National Institutes of Health. This means that study information cannot be given as part of any legal actions or court cases unless you give your assent. However, we *do* have to follow laws that would require us to report certain kinds of information, like when there is child abuse.<br><br>If you are asked to complete the CRAFFT Interactive, your doctor will see those responses. However, your doctor and your parents/guardians will not see any of your survey answers, unless any of your answers make us worried about your immediate safety. If that happens, we will tell your doctor about our concern.<br><br>Do you have any questions so far?<br>...
**REDCap/Twilio**	Throughout the study, we'll use a program called REDCap to securely collect and store your survey answers. We'll also use a third-party service called Twilio to send you text or email	Some other things that you should know about are how we collect and store your data, and who may see it. We use a program called REDCap for securely collecting and storing your survey answers using password protection.

	notifications about new surveys to complete.<br><br>You don't need to download any apps to use these programs. Both of them are designed to keep your information safe and private.<br><br>Do you have any questions about REDCap or Twilio?	To send you text-message or email reminders in this study, we use a third-party service called Twilio. Twilio does store your phone number and email address in their private logs, and, because Twilio is separate from Boston Children's Hospital, we do not have the ability to delete that information. Neither we nor Twilio will ever contact you about things that aren't related to the study.<br><br>You don't need to download any apps to use these programs. Both of them are designed to keep your information safe and private.
**Data Sharing**	Information that we collect from you may be viewed by people who oversee this study. Unless there is a legal reason, this data will be "de-identified." That means that all personal identifying information about you has been removed. Eventually, once we finish the study, we will analyze the de-identified data of everyone in the study.<br><br>*Also, de-identified data might be shared in a large database that helps researchers, called the NIAAA Data Archive. It is your choice whether to have your data be included in this database. You can still be in this research study even if you decide that you do not want your data to be added to the NIAAA data archive*<br><br>Do you have any questions about the data we collect from you, or how it's used or shared? …	You should also know that any information we collect from you may be viewed by people who oversee this study to make sure it's being done correctly. Unless there is a legal reason, this data will be "de-identified." That means that your name and other information that could identify you is removed.<br><br>Also, de-identified data might be shared in a large database that helps researchers, called the NIAAA Data Archive. There is a chance that someone will use this data to try and learn your identity, but this risk is very small. It is your choice whether to have your data be included in this database. You can change your mind later on, but we won't be able take back any data that has already been shared. *You can still be in this research study even if you decide that you do not want your data to be added to the NIAAA data archive*<br><br>When we look at the study results, the data will be de-identified. We will share a summary of our de-identified study results on a website called clinicaltrials.gov, as required by U.S. law. We may also share the de-identified data with other researchers.

<table>
<tr><td></td><td></td><td>Do you have any questions about the data we collect from you, or how it's used or shared? …</td></tr>
<tr><td>Summary of Rights</td><td>Finally, as a reminder, joining this study is completely up to you. You don't have to join if you don't want to. If you join, you can decide to stop at any time.</td><td>Finally, you should also know that the researchers might decide to take you off the study early, like if we feel that it is in your best interest to stop, or if we stop the study for everybody.

As a reminder, joining this study is completely up to you. You don't have to join if you don't want to. If you join, you can decide to stop at any time.</td></tr>
<tr><td>Transition to Comprehension Questions</td><td colspan="2">So, I know I've given you a lot of information! Do you have any questions?
…

To make sure we're on the same page, I'd like to ask you a few questions that check your understanding of what I explained. I'll read a statement, and you can tell me whether it's true or false. Ready?

A. Being in this study is completely up to you, and whether you join or not will not affect the care that you receive at your doctor's office, now or in the future. (TRUE / FALSE)
• IF ANSWERED INCORRECTLY (said FALSE): This statement is actually true! Your participation is completely up to you, and will not impact the care you receive, now or in the future. Do you have any questions about this?
…
So, I'll ask again…[READMINISTER QUESTION.]
B. If you join this study, you can still decide to leave at any time. (TRUE / FALSE)
• IF ANSWERED INCORRECTLY (said FALSE): This one is also true! You can choose to leave at any time. Do you have any questions about this?
...
Just to check, I'll ask again… [READMINISTER QUESTION.]
C. Your name will never be stored with the answers that you give on surveys that you fill out. (TRUE / FALSE)
• IF ANSWERED INCORRECTLY (said FALSE): This one's true! Your name will never be attached to any of the surveys that you complete. If you are asked to do the CRAFFT Interactive, your doctor will know which answers are yours, but we will never store your name in the same place as your answers. Do you have any questions about this?
...
Just to check, I'll ask again… [READMINISTER QUESTION.]</td></tr>
</table>

<table>
<tr>
<td rowspan="2"></td>
<td colspan="2">
D. Your parents/guardians will be told what you answered on the surveys. (TRUE / <u>**FALSE**</u>)
• **IF ANSWERED INCORRECTLY** *(said TRUE):* This one is false. Your survey answers are confidential. We will not share any of your answers with your parents, unless your doctor thinks you're at risk for hurting yourself or someone else. In that case, they'd talk to you first about what information to share with your parents. Do you have any questions about this?
...
Just to check, I'll ask again… [READMINISTER QUESTION.]
E. If we see something in your survey answers that make us worried about your immediate safety, we will let your doctor know. (<u>**TRUE**</u> / FALSE)
• **IF ANSWERED INCORRECTLY** *(said FALSE):* This statement is true, and it's because we want to make sure that if something in your survey answers makes us worried about your immediate safety or mental health, we want your doctor to help address it. They will discuss this concern with you before sharing anything with your parent or guardian. Do you have any questions about this?
...
Just to check, I'll ask again… [READMINISTER QUESTION.]
F. A risk of participating is that there is a small chance that someone that is not part of the study could learn your survey answers. (<u>**TRUE**</u> / FALSE)
• **IF ANSWERED INCORRECTLY** *(said FALSE):* This one is also true. There is a very small chance that someone that is not part of the study could learn your survey answers. This is why we won't collect your name on any surveys you fill out for the study. Do you have any questions about this?
...
Just to check, I'll ask again… [READMINISTER QUESTION.]

Great, thanks so much for going through these questions with me.
</td>
</tr>
<tr>
<td>**IF, AFTER 2 TRIES, 6/6 ANSWERED CORRECTLY:** CONTINUE TO "ASSENT"]</td>
<td>**IF, AFTER 2 TRIES, ANY ARE STILL ANSWERED INCORRECTLY]** Based on your answers, it appears that this study may not be the best fit for you to join. But, I really appreciate your time today. Have a good rest of your day!

[RA ENDS CALL]</td>
</tr>
<tr>
<td>**Assent**</td>
<td colspan="2">It is now time to decide whether you would like to join this study. You can check in with your parent or guardian first, if you would like.

[LET TEEN CHECK IN WITH PARENT IF DESIRED].</td>
</tr>
</table>

<table>
<tr>
<td rowspan="2"></td>
<td colspan="2">Do you agree to participate in this study?

[RA DOCUMENTS ANSWER IN REDCap]</td>
</tr>
<tr>
<td>***[IF YES]*** Wonderful!

↳ **[IF TEEN IS TURNING 18 IN NEXT ~14 MONTHS]** I see that you will turn 18 during your time in this study. So, when you turn 18 – meaning you are considered an "adult" – we'll check in with you around your 18th birthday to check if you still want to be in the study. We'll text or email you a 1-question response form where you can tell us your choice to continue or not. You'll need to answer this question before continuing with any more study activities. Do you have any questions about that?... OK, let's keep going. I have a few more questions for you, I'll have you test out your ability to receive our notifications, and then we're done.

↳ **[IF NOT TURNING 18]** I have a few more questions for you, I'll have you test out your ability to receive our notifications, and then we're done.</td>
<td>**[IF NO]** That's okay, I really appreciate your time today. Would you mind sharing your reason why not?
...
Ok, thanks so much for speaking with me today. Have a good rest of your day!

[RA RECORDS REASON FOR DECLINE & END CALL]</td>
</tr>
<tr>
<td rowspan="2">**NIAAA Data Archive Permission/ GUID Creation**</td>
<td colspan="2">Do you also give permission for your de-identified data to be shared with the large database at NIAAA?

[RA RECORDS ALL ANSWER IN REDCap]</td>
</tr>
<tr>
<td>***[IF YES]*** Thank you for giving your permission. We now need to collect some information from you. The info we collect here is <u>not</u> shared with the NIAAA data archive. Instead, this info will help us create your unique ID number that we will use to submit data to the NIAAA data archive.

• Recognizing that you might not use this name, what is your first, middle, and last name that appears on your birth certificate?

• In what city were you born? This is the city that appears on your birth certificate.</td>
<td>***[IF NO, [RA CONTINUES TO CONTACT SURVEY, PLEASE SEE ATTACHMENT M.2 BEFORE RETURNING TO THE BELOW SCRIPT. THIS SURVEY WILL BE 2-5 MINUTES AND WILL BE CONDUCTED OVER THE PHONE IN THIS SAME CALL]***</td>
</tr>
</table>

	[RA CONTINUES TO CONTACT SURVEY; PLEASE SEE ATTACHMENT M.2 BEFORE RETURNING TO THE BELOW SCRIPT. THIS SURVEY WILL BE 2-5 MINUTES AND WILL BE CONDUCTED OVER THE PHONE IN THIS SAME CALL]	
**Contact for Future Studies**	There may be future adolescent health research studies that we would like to contact you about, if you give us your permission. For example, we may want to send you additional paid follow-up surveys in future years, after this study is completed. Your participation in any future research is completely up to you.<br><br>Would you be willing to have us contact you in the future about participating in other research studies on adolescent and young adult health?<br>…<br>***[RA RECORD ANSWER IN REDCap]***	
**Test for receiving notifications**	Thanks! We're really close to wrapping up. The last thing we need to do is make sure you're set up to use the online surveys and survey notifications.<br><br>First, I want to make sure you're able to receive our notifications. I'm going to [TEXT/EMAIL] you a word.<br><br>***[RA SENDs TEXT/EMAIL CONTAINING "START" VIA TWILIO]***<br><br>What is the key word you just received? …Great, I'm glad you got that.<br><br>➔ **[IF EMAIL PREFERENCE:** ***IF WORD NOT RECEIVED WITHIN A FEW MINUTES, ASK TO TRY TEXT. IF SUCCESSFUL, CONTINUE TO PASSWORD SETUP*****]**<br><br>➔ **[IF TEXT MESSAGE PREFERENCE:** ***COMPLETE OPT-IN PROCEDURE TO PREVENT CARRIER SPAM FILTERING*****]** Now, can you please send a reply to that message with the same word, START? Replying with this word will help your cell phone carrier know that our texts aren't spam messages. Have you sent the word START?...Thanks! Just so you know, all future text messages from us will be automated, and we are unable to see anything you might reply. So, please do not reply to future text messages. Instead, please contact us by phone call with any questions that come up for you.<br>• ***[IF WORD NOT RECEIVED WITHIN A FEW MINUTES, ASK TO TRY EMAIL]***<br>• ***[IF SUCCESSFUL, CONTINUE TO PASSWORD SETUP]***	
**Password Setup**	Now that I know you can receive notifications, I am going to send you a link where you can set your password for the surveys. This should be a password that you don't use for anything else, only for ASPIRE. I would recommend writing it down somewhere private, so you don't forget. I'll stay on the phone while you're setting it.<br><br>***[RA SENDS PASSWORD RESET SURVEY, CONFIRMS COMPLETION BY CHECKING REDCAP RECORD]***	

**Copy of Participant Info Sheet**	All set? Great. I know we went over a lot of information today! So, again, I want to let you know that everything we talked about is also in writing, in an information sheet. ***[IF PARTICIPANT COMPLETED ONLINE:*** This was the document that appeared at the end of the survey you took.]<br><br>We send a copy of this information sheet to everyone who joins the study. That way, if you think of any questions after we hang up, the sheet might be able to tell you the answer.<br><br>Would you prefer the information sheet be sent to you by email, or to your home as a paper copy?<br><br>• ***[IF EMAIL, VERIFY THE EMAIL ADDRESS]***<br><br>• ***[IF PAPER COPY MAILED TO HOME, VERIFY THE HOME ADDRESS]***
**Wrap-up**	After we get off this call today, if you have any more questions or concerns about the study, you can get in touch with our study team or the IRB administrator. Our contact information is listed in the study flyer you got in the mail, and in the Information Sheet you're receiving.<br><br>Thank you so much for your willingness to participate in this important study. We'll be in touch with the link to your first survey. Thanks, and have a great day!<br><br>***[END CALL]***
