# Supplementary material for: Computer-Facilitated Screening and Brief Intervention for Alcohol Use Risk in Adolescent Patients of Pediatric Primary Care Offices: Protocol for a Cluster Randomized Controlled Trial
Source: JMIR Res Protoc. 2024 Mar 26;13:e55039. doi: 10.2196/55039 (PMC11005433; doi:10.2196/55039)
Supplement: Multimedia Appendix 3 [file resprot_v13i1e55039_app3.pdf]

SUMMARY STATEMENT
(Privileged Communication)

PROGRAM CONTACT:
Lori Ducharme
301-451-8507
lori.ducharme@nih.gov

Release Date: 07/01/2019
Revised Date:

Application Number: 1 R01 AA027253-01A1

Principal Investigator

HARRIS, SION KIM

Applicant Organization: BOSTON CHILDREN'S HOSPITAL

Review Group: AA-3
Clinical, Treatment and Health Services Research Review Subcommittee

Meeting Date: 06/21/2019
Council: OCT 2019
Requested Start: 09/01/2019
RFA/PA: PA19-055
PCC: AS L

Project Title: Computer-facilitated Screening and Brief Intervention in pediatric primary care to reduce underage drinking: a large multi-site randomized trial
SRG Action: Impact Score:34 Percentile:19 #
Next Steps: Visit https://grants.nih.gov/grants/next_steps.htm
Human Subjects: 30-Human subjects involved - Certified, no SRG concerns
Animal Subjects: 10-No live vertebrate animals involved for competing appl.
Gender: 1A-Both genders, scientifically acceptable
Minority: 1A-Minorities and non-minorities, scientifically acceptable
Age: 2A-Only Children, scientifically acceptable

Project Year	Direct Costs Requested	Estimated Total Cost
1	427,735	724,213
2	436,145	738,452
3	439,471	744,083
4	441,317	747,209
5	352,167	596,266
TOTAL	2,096,835	3,550,223

ADMINISTRATIVE BUDGET NOTE: The budget shown is the requested budget and has not been adjusted to reflect any recommendations made by reviewers. If an award is planned, the costs will be calculated by Institute grants management staff based on the recommendations outlined below in the COMMITTEE BUDGET RECOMMENDATIONS section.
NEW INVESTIGATOR

1R01AA027253-01A1 Harris, Sion

NEW INVESTIGATOR
RESOURCE SHARING PLANS: UNACCEPTABLE

RESUME AND SUMMARY OF DISCUSSION: This is an amended R01 submission proposing a multisite study comparing computerized SBI to standard care in a large and diverse sample of adolescents (aged 14-17) in a range of outpatient pediatric primary care settings associated with the American Association of Pediatrics (AAP) research network. The resubmitted application retains previously noted strengths - an outstanding research team, access to a nationwide network which would provide access a large sample size, and a strong environment, design features such as the inclusion of a TAU control, a one-year follow-up and the use of AAP network for dissemination. The applicants have been responsive and have satisfactorily addressed most of the methodological concerns. Concerns remain regarding innovation, and the approach as proposed still appears to yield information that is somewhat incremental, only adding a few new features, rather than provide a 'definitive' study of cSBI for adolescents. Nevertheless, proposed studies are significant and there was a strong enthusiasm for this application.

DESCRIPTION: Alcohol use disorders frequently have a pediatric origin. Pediatric primary care offices, where the majority of adolescents receive health care, are a promising venue for early identification and intervention through universal screening and brief counseling. However, while the U.S. Preventive Services Task Force recommends primary care-based alcohol screening and brief intervention for adults, it found insufficient evidence to recommend it for adolescents. The goal of the proposed study is to address this evidence gap by testing the effectiveness of a promising computer-facilitated Screening and Brief Intervention (cSBI) system for delivery by pediatric primary care clinicians to adolescents at well-visits. This cluster-randomized controlled trial will be conducted in the American Academy of Pediatrics' (AAP) Pediatric Research in Office Settings (PROS) national primary care practice-based research network. PROS has demonstrated success in >30 years of practice-based research, with >600 practices participating in recent studies. Drawing on more than 15 years of our prior research on adolescent alcohol screening and brief counseling in primary care offices, the cSBI system was developed to provide a time-efficient and feasible way for pediatric practices to improve both the frequency and quality of alcohol screening and counseling. cSBI includes: 1) computer self-administered screening that adolescents complete prior to seeing their clinician, 2) computer-delivered personalized feedback to the adolescent, 3) 10 interactive psychoeducational pages for the adolescent on substance use health risks, 4) a Clinician Report Form with screen results and prompts that clinicians use to provide motivational interviewing-based individualized counseling, and 5) clinician training materials and protocol. In our New England-based pilot study, we found that, compared to usual care (UC), the cSBI approach: 1) increased patient receipt of alcohol-related counseling during well-visits; 2) improved patients' ratings of the quality of their clinician encounter; and 3) among those who reported past-year use at baseline (n=192), was associated with a 34% lower risk of a heavy episodic drinking episode during 12 months follow-up. cSBI also reduced risk of riding with an impaired driver, a major safety risk associated with alcohol, by 42% among those with prior riding risk. A larger RCT of this approach, which employs an adequately powered sample and tests generalizability of effects beyond New England, is needed. We propose to randomize >30 pediatric primary care clinicians in 10 practices nationwide to deliver UC or cSBI (1:1) to their eligible and assenting 14- to 17-year-old patients arriving for well-visits. Our aims are to test cSBI's effect on heavy episodic drinking, and on riding with an impaired driver/driving while impaired, during 12 months follow-up among 1,268 adolescents screen-identified as at-risk. If effective, cSBI dissemination could leverage existing AAP platforms including education, teaching, and advocacy, to its 67,000 pediatrician members, greatly increasing the potential for population-level impact of alcohol screening and brief intervention for U.S. adolescents.

PUBLIC HEALTH RELEVANCE

Our goal is to conduct a large multi-site randomized controlled trial (RCT) of a promising computer-facilitated Screening and clinician Brief Intervention (cSBI) system designed for delivery by pediatric primary care clinicians and aimed at reducing unhealthy alcohol use and related riding/driving safety risk among adolescent patients. Our setting will be the American Academy of Pediatrics' (AAP) Pediatric Research in Office Settings (PROS) national primary care research network, with U.S. primary care practices having participated in recent studies. This trial addresses the evidence gap identified in the latest U.S. Preventive Services Task Force review of alcohol screening and brief counseling interventions among adolescents, and, if shown effective, the cSBI system could be widely disseminated via AAP's existing education, teaching, and advocacy platforms to its 67,000 pediatrician members, thereby greatly increasing the potential for population- level impact of alcohol screening and brief intervention for U.S. adolescents.

CRITIQUE 1

Significance: 3
Investigator(s): 2
Innovation: 4
Approach: 4
Environment: 1

Overall Impact:

This is a resubmission of a multisite study to evaluate computerized SBI to standard care in a large and diverse sample of adolescents (aged 14-17) in a range of outpatient pediatric primary care settings associated with the AAP research network. In the previous reviews, strengths included an outstanding team, access to a nationwide network which would provide access a large sample size, and a strong environment. Inclusion of a TAU control and a one-year follow-up were also strengths, as would be the AAP network for dissemination.

Weaknesses identified in the previous reviews were multiple and significant. These included lack of innovation regarding E-SBI, the multi-component approach to be evaluated with several untested components included, lack of rationale for cannabis use as a secondary aim, questions about incremental gains to knowledge for a fairly well-studied area, and whether the modest effects of the previous study by this team justified a large, multisite trial. Reviewers pointed out that evaluation of long-term harms would be needed generate data that would affect policy. The texting component was not well developed or integrated. There were also major concerns about plans to deal with contamination. Practical issues noted included the need for more attention to fidelity ratings and training of staff in multiple sites.

The resubmission was responsive to many of the issues raised in the prior review. The start-up period was shortened, the untested aspects of the intervention were dropped, randomization would be via clinician rather than patient to reduce contamination. The investigators clarified that AAP policy is that network sites can only be selected after a project is funded and IRB approval is in place; the PROS network is established and has its own single IRB, another plus. The waiver rather than parental consent appears justified given the low risk and the nature of this study. The project, following advice from reviewers, is now better positioned to address USPSTF issues related to SBI in adolescents, specifically around drinking related risks and harms in this population. The intervention itself is simpler and less of a "kitchen sink" approach.

However, the issues of innovation and incremental value to the data to be gained were not fully addressed. The large study evaluating the cSBI intervention is still under review; the data presented in the application was not particularly compelling and there was no strategy given for strengthening the approach. Fidelity evaluation remained weak. Outcomes would be entirely based on self-report. No

cost-effectiveness aspect was included. Hence, the approach still appears somewhat incremental, only adding a few new features, rather than a 'definitive' study of cSBI for adolescents.

1. Significance:

Strengths

- As before, using pediatric primary care visits as a conduit to prevent or reduce risky alcohol in adolescents is of high public health significance.
- As before, universal screening regardless of drinking status adds impact; however
- As before, delivering via computer should facilitate dissemination if the approach demonstrates significant effects on drinking, health, and risk outcomes.

Weaknesses

- As noted above, the previous, similar, large multisite study of the approach remains under review. The investigators did not make a strong case regarding important novel aspects of this work.

2. Investigator(s):

Strengths

- As before, the investigative team is strong and clearly capable of carrying out the proposed work.
- Dr. Harris is a productive young investigator with almost 80 peer reviewed publications, most in areas related to the proposed work.

Weaknesses

- No concerns.

3. Innovation:

Strengths

- Evaluation of SBI is not particularly innovative and there are several studies in this area. The cSBI approach to be evaluated here has been fairly well studied before, with modest effects.

Weaknesses

- None noted.

4. Approach:

Strengths

- As before, the practice care network, cSBI approach, follow-up, sample size, and other aspects noted are all strengths that still pertain. The sample (1268 adolescents aged 14-17 in diverse areas across the US) ample, and large and diverse enough for generalizability.
- Retention rates in prior similar studies by this group have been good (approaching 80%).
- The cSBI intervention feedback and brief interventions are somewhat tailored to the adolescents' reported drinking level.

- Clinicians will be randomized within practice; again, however, since SBIRT activities have been around for many years and are widely disseminated and recommended by AAP, its likely that many of the clinicians not randomized to cSBI will be doing a reasonably good job of screening and intervening with adolescents at risk, reducing likelihood of a larger effect.
- Training of the clinicians assigned to the cSBI approach is reasonable without being overly burdensome.

Weaknesses

- The time to drinking data presented in Figure 2 did not indicate a significant effect, leading to questions about the strength and promise of the cSBI intervention. Again, since the main report remains under review and is not available to reviewers, the present studies may be premature and justification was not compelling.
- The investigators appear to be moving away from universal screening by including adolescents with past-year drinking or riding in a car with an impaired driver, which may reduce generalizability.
- The fidelity rating plan is improved but remains fairly passive and not consistent with standard methods of assessing fidelity to behavioral interventions. The rating form to be used was not specified and it was somewhat surprising the investigators had not developed this in prior research.
- Outcomes will be entirely based on self-report rather than medical records. The rationale for not collecting biological samples was not compelling and a major weakness that could undermine credibility of findings and their persuasiveness to the USPSTF.
- The statistical plan does not consider the possibility of interventionist (clinician) effects.
- It was not clear why a cost-effectiveness analysis was not involved, again reducing likely impact of results.

5. Environment:

Strengths

- As before, the environment is strong, no concerns.

Weaknesses

- None noted.

Study Timeline:

Strengths

- Seems reasonable, if ambitious. Long lead in period was reduced.

Weaknesses

- No concerns.

Protections for Human Subjects:

Acceptable Risks and/or Adequate Protections

- As before, it is clear that the team has experience to conduct this trial safely with multiple subject protections in place. Using the single IRB associated with the AAP network is a plus.

Data and Safety Monitoring Plan (Applicable for Clinical Trials Only):

Acceptable

Inclusion Plans:

- Sex/Gender: Distribution justified scientifically
- Race/Ethnicity: Distribution justified scientifically
- For NIH-Defined Phase III trials, Plans for valid design and analysis: Not applicable
- Inclusion/Exclusion Based on Age: Distribution justified scientifically

Resource Sharing Plans:

Acceptable

Budget and Period of Support:

Recommend as Requested

CRITIQUE 2

Significance: 4
Investigator(s): 2
Innovation: 5
Approach: 5
Environment: 1

Overall Impact:

This revised proposal from an NIH NEW INVESTIGATOR is for a cluster-randomized efficacy trial of pediatricians, testing a computerized alcohol screening for alcohol use, followed by computerized brief education (cSBI) followed by pediatrician brief advice. The proposal explicitly targets the USPSTF call for more research on alcohol SBI in adolescents. The trial (in 10 PROS practices) evaluates the intervention in an estimated 1268 "at-risk" patients between ages 14-17 seeing 30 pediatricians who enroll and who themselves consent. Patient eligibility is determined based on annual well visit, screening positive for past 12-month alcohol use, or riding risk, and having cell phone. The trial is generally rigorously designed, and the revision is responsive to many prior critiques. Issues from the prior reviews that were addressed include: a conceptual model, omission of add-on texting interventions, how MI is incorporated, explanation of training of the pediatricians and rigorous monitoring of fidelity. The revision also explains that the prior "trial" was actually a pre-post study with major flaws. A waiver of consent from patients appears to be feasible.

Major limitations of the current proposal, however, persist. Despite saying that the USPSTF is a major target audience of this trial, the trial does not address why the PIs prior trial was not included in the recent USPSTF review. It appears it was deemed "prevention" because the sample (apparently all pediatric patients) was not of interest to USPSTF. The current proposal focuses on patients who have used any alcohol in the past year, but the outcome is heavy episodic drinking (any). It is unclear that this sample will be acceptable to USPSTF and that is not addressed. No data in the Approach section indicates this is an adequate screen for unhealthy alcohol use –the NIAAA screen is noted to be a valid screen for any use past year (page 116). This could still be perceived as prevention by USPSTF, especially since the outcome is not any use. While trials of BIs are needed with adolescents with unhealthy alcohol use, this proposal does not clearly demonstrate that it fills that gap.

1. Significance:

Strengths

- Unhealthy alcohol use in youth is associated with marked adverse impacts on like and heath
- USPSTF has highlighted the need for high quality trials of youth alcohol SBI in primary care
- Evidence suggests that SBI might be efficacious.
- Computerized screening has advantages in terms of privacy and workflow
- PI's group previously conducted a pre-post "trial" with 18 months of pre, no apparent washout (to avoid intervention after conversion of the clinic), exclusion of patients from the post period (resulting in differences in the 2 samples in terms of drinking), and evaluating drinking at 3 and 12 months (not HED). Certainly, that is not a rigorous design, suggesting that further study is warranted.
- Premise: Need for high quality studies is strong.
- References to USPSTF calls for more research (KQ#4 and KQ#5) are helpful.

Weaknesses

- Literature and preliminary studies review unclear and deficient. This reviewer had to review the Pediatrics 2012 manuscript to understand the above, indicating that the proposal did not still delineate characteristics of the prior studies and the proposed study and the 2 trials reviewed by USPSTF in 2018. A table of sample, intervention, outcomes and follow-up would have helped understand what USPSTF requires.
- Specifically, the limitations in the extant literature would ideally have been much more clearly summarized, including the USPSTF review (2 studies) and trials since. Only 2 trials met quality criteria for the USPSTF review 2018 which admittedly came out several months before this proposal was submitted. It would have been helpful to clearly state how the proposed trial differed and how it was the same as those reviewed and those excluded. (Mason M, Light J, Campbell L, et al. Peer Network Counseling with Urban Adolescents: A Randomized Controlled Trial with Moderate Substance Users. J Subst Abuse Treat. 2015;58:16-24; Haug S, Paz Castro R, Kowatsch T, et al. Efficacy of a Web- and Text Messaging-Based Intervention to Reduce Problem Drinking in Adolescents: Results of a Cluster-Randomized Controlled Trial. J Consult Clin Psychol. 2016.)
- **Primary Prevention Trials not of interest to USPSTF**. The prior trial by the PI (Pediatrics 2012) was not included in the USPSTF review (reason "prevention"). It would have been helpful if the proposal delineated whether the proposed trial was designed in a manner where it would have overcome limitations of the prior study and been eligible for inclusion in the 2018 review. Any alcohol use past year seems like primary prevention to this reviewer given that the outcome is heavy episodic drinking. Page 12 clearly cites USPSTF interest in interventions that "reduce unhealthy alcohol use". The decision to define any past year use as unhealthy alcohol use might have been considered acceptable if the trial used that as an outcome. However, this reviewer does not expect most experts would define unhealthy alcohol use as any past year use (or at least it would need to be clear whether or not that has not been the gold standard for unhealthy alcohol use in validation studies of alcohol screens for unhealthy alcohol use in adolescents).

2. Investigator(s):

Strengths

- Dr. Harris (PhD Johns Hopkins 1996), Assoc. Professor Pediatrics Harvard Medical School (2017-), and Director of CeASAR, Boston Children's Hospital since 2018 when Dr. Knight

retired. She is a new NIH investigator, having been made a MPI after submission for the prior study.

- Dr. Burke (Northeastern U Clinical Professor since 2014 and PhD Program Director and MINT) brings MI expertise and will provide clinician trainings. She has done the trainings for a grant with Sharon Levy and Eli Weitzman, as well as the Moment study (Shrier PI)
- Dr. Fiks (Assoc. Professor U Penn) is a practicing pediatrician and Director of the American Academy of Pediatrics (AAPs) Pediatric Research in Office Settings (PROS) Research Network, who has years of experienced in practice-based research including meading or MPIing R01s and P30s. Dr. Fiks will lead the PROS team contributing to design, implementation, analysis and manuscript preparation.
- Dr. Knight (developer of the CRAFT and founder of CeASAR) retired 12/31/2017 but will be a consultant (25-100 hours a year) to support Dr. Harris.
- Lon Sherritt MPH (1999) has designed and conducted analyses as well as web platforms and NLP algorithms for CeASAR since its inception,
- Dr. Shone (PhD, MSW) is the Director of Primary Care Research for the AAP and will oversee the trial for AAP.

Weaknesses

- Minor: While several investigators have experience with clinical trials, there is no clear trialist on the team. While the generally strong protocol suggests that there might be adequate expertise, this reviewer would like to see a PhD-level statistician with experience in pediatric trials.

3. Innovation:

Strengths

- Use of AAP PROS for alcohol research is novel

Weaknesses

- This is incremental research that does not address the challenge of unhealthy alcohol use in adolescents. Although it is sorely needed. All elements have been studied in prior studies by the PIs team—albeit with inadequate rigor.

4. Approach:

Strengths

- Strong intervention. Combining technology for screening and psycho-ed feedback, with primary care clinicians' advice, seems like a strength.
- The trial is rigorously designed and described including recruitment, the intervention, rationale for waiver of parental consent,
- While USPSTF has relied on consumption outcomes for adults, measures of harms are encouraged, and have been added.

Weaknesses

- Sample. Including patients who only used 1 or more times in the past year (irrespective of age) will make it hard to show benefit (see figure on consumption and outcomes in the USPSTF 2018). No evidence is presented that this is a validated screen for unhealthy alcohol use. That could also make the USPSTF reviewers consider it primary prevention (which appears to make them omit the PIs study in Pediatrics in 2012). Screening for unhealthy alcohol use and/or AUDs is strongly advised if the goal is to decrease heavy drinking at 12 months follow-up. Also,

no preliminary data are recalled by this reviewer to demonstrate high rates of heavy episodic drinking during follow-up among those with any past 12-month use.

- Preliminary studies of the intervention (cSBI) were described as an RCT that was really a pre-post evaluation. It did not find cSBI efficacious (pre-post). That study had a number of limitations: patients in phase 1 (UC) were removed from phase 2 (intervention) and there were significant differences between the groups, as well as lack of a washout period between the 18 months. A quick read did not identify any randomization (as in a stepped wedge design).
- Measures. Measuring DSM-5 AUD symptoms at baseline would be critically important. Outcomes may vary depending on AUD v not.
- This reviewer would have liked to have a clear statement that the trial was addressing and meeting USPSTF criteria for the recent 2018 review on alcohol interventions. It appears it would not have.
- Outcomes: Objective outcome measures are critically important. Relying on patient self-report allows social desirability bias to impact the trial. Perhaps change in grades could be added as a secondary outcome?

5. Environment:

Strengths

- CeASAR has a long track record of research in this area.
- The APP PROS has a track record for office-based trials.

Weaknesses

- None noted.

Study Timeline:

Strengths

- Acceptable

Weaknesses

- No limitations noted

Protections for Human Subjects:

Acceptable Risks and/or Adequate Protections

- Obtaining central IRB (AAPs IRB) approval would help reviewers know that a waiver of parental consent is possible

Data and Safety Monitoring Plan (Applicable for Clinical Trials Only):

Acceptable

Inclusion Plans:

- Sex/Gender: Distribution justified scientifically
- Race/Ethnicity: Distribution justified scientifically
- For NIH-Defined Phase III trials, Plans for valid design and analysis: Not applicable
- Inclusion/Exclusion Based on Age: Distribution justified scientifically

Revision:

- Limitations - see above

Budget and Period of Support:

Recommend as Requested

CRITIQUE 3

Significance: 3
Investigator(s): 2
Innovation: 3
Approach: 3
Environment: 1

Overall Impact:

The randomized clinical trial proposed in this revised application builds on prior work that showed a multi-faceted computerized screening tool paired with direct face-to-face counseling delivered in pediatric primary care offices reduced heavy episodic drinking among adolescents who reported past-year alcohol use at baseline. This proposal aims to replicate the initial study in a larger sample that is geographically and socioeconomically diverse. Identifying promising new ways to reach adolescents, an especially vulnerable population in the context of alcohol use and misuse, could have significant clinical and public health implications. The prior submission was considered meritorious based on compelling preliminary data that supported the feasibility and efficacy of the approach, the aim to leverage pediatric practices as a natural point of engagement for youth, the careful selection of measures, and the overall study design. Prior concerns were related to the unconvincing justification for a second large-scale randomized clinical trial that would essentially replicate recent work by the investigative team. In addition, there were concerns that this new trial would still prove insufficient to move the US Preventative Task Force to recommend adolescent screening and brief intervention (a stated goal of the applicants). Additional questions were raised regarding the multiple untested additions to the initial brief intervention (especially the texting protocol), clinician training and fidelity monitoring, the scope and feasibility of the proposed work given that specific clinic sites were not identified and the staffing plan seemed logistically challenging, and insufficient attention to health-risks associated with adolescent drinking. Many of these concerns were carefully considered and sufficiently addressed in the revised application. Concerns remain, however, about the justification for a second large-scale trial. In addition, it is unclear how preliminary findings relate to the proposed work given that participants in the initial trial were required to report past 12-month drinking at baseline. The proposed study will enroll nondrinkers along with drinkers and inadequate attention is given to how these two distinct groups of youth will be handled in analyses. In addition, inasmuch as the proposed computerized screening and in-person brief intervention requires considerable time, there is question about how well this approach – even if efficacious – will translate to typical pediatrician practices. Indeed, the proposed approach requires clinicians to complete several hours of intensive training, establish baseline competency, and engage in ongoing adherence monitoring and expert feedback. On the whole, however, the investigators were responsive to prior critiques, the proposed work could have substantive impact clinical care for adolescents, and there is no question the investigative team could successfully execute the proposed research.

1. Significance:

Strengths

- Alcohol use typically begins and escalates during adolescence, and the onset of alcohol-related problems often occurs during the teenage years. Advancing and streamlining screening and brief intervention approaches during this developmental period holds promise for prevention and early intervention.
- Integrating alcohol use screening during routine appointments with pediatricians capitalizes on an opportune point of engagement, at least for many youths. This approach would afford a notable advancement over (or least an important complement to) prior work, which largely focused on emergency departments and school settings.
- Initial data demonstrate considerable promise for the proposed approach.
- The proposed work would leverage a multi-faceted computerized screening tool paired with direct face-to-face counseling to advance both prevention (among nondrinkers) and intervention efforts. This is a notable strength that adds considerable significance.

Weaknesses

- As noted in the prior review, the investigative team recently completed a large-scale multisite study to evaluate the feasibility and efficacy of the proposed screening and prevention/intervention approach. Results supported the approach. It remains unclear whether replicating the initial trial would yield value-added information beyond what is already known. With that being said, replication is an important scientific endeavor and would afford increased confidence in the impact of this screening and brief intervention approach and allow for better generalization to the larger population of youth. These advances may be important (and ultimately necessary) to influence pediatrician practices to implement this somewhat time-intensive intervention.
- Prior concerns were related to whether this new trial would prove insufficient to move the US Preventative Task Force to recommend adolescent screening and brief intervention, a stated goal of the applicants. This applicant did not address this concern in the revised application.

2. Investigator(s):

Strengths

- The principal investigator, Dr. Sion Kim Harris, is a promising new investigator who has the requisite expertise to successfully execute the proposed work. Dr. Harris successfully completed a multisite project that tested the proposed computerized screener/brief intervention in primary care settings. She has demonstrated an ongoing record of scholarly accomplishments in areas directly related to proposed research.
- Dr. Harris is supported by a team of established and productive co-investigators.

Weaknesses

- Although Dr. Harris’ biosketch states she will serve as MPI on this application, this seems inaccurate given there is no other mention of this role. This seems to be a typographical error and thus only a very minor weakness.

3. Innovation:

Strengths

- Leveraging pediatric practices as a natural point of engagement for youth is innovative.

Weaknesses

- Inasmuch as this proposal is designed to replicate the applicants recently completed clinical trial of the same intervention, the proposed work is not innovative.
- Computerized screening tools for alcohol use are neither new nor innovative.

4. Approach:

Strengths

- Consideration of multiple outcomes, including alcohol use, alcohol-related problems, and other risk behaviors, is a strength of the application.
- The proposed work builds on strong initial data (*N* = 192) with high 12-month retention rates (80%), which supports the promise of the approach along with the feasibility of successfully executing the proposed large-scale project.
- The focus on enrolling a geographically, socioeconomically, and racially diverse sample is an important strength of the proposed work. In the preliminary study, approximately half (51%) of the participants were female and half were ethnic or racial minorities (55%), which provides confidence that findings from the proposed work would generalize to a broad range of youth.
- The sizable sample (N = 1,268) is a notable strength, and the applicants' track record of recruiting large numbers of youth demonstrates the feasibility of meeting this recruitment target.
- Providing adolescents with personalized feedback tailored to their level of alcohol involvement is a strength.
- The intensive training and adherence monitoring provided to clinicians is a strength. On balance, however, this approach raises some concern about the ultimate utility of this approach, even if it demonstrates efficacy.

Weaknesses

- A common barrier pediatricians confront when it comes to alcohol screening is how to ethically and adequately manage adolescents who report risky and problematic alcohol use. The application was virtually silent regarding how this important obstacle would be addressed. What support or guidance will pediatricians (or other healthcare workers in pediatric offices) receive regarding youth who report clinically significant alcohol or drug problems? Will parents be notified? Additional details are needed regarding these important considerations.
- It is unclear how preliminary findings relate to the proposed work given that participants in the initial trial (or at least included in the reported analyses) were required to report past 12-month drinking at baseline. The proposed study will enroll nondrinkers (along with drinkers).
- Primary outcomes focus on between-group differences in alcohol use and alcohol-related problems. But nondrinkers are eligible to participate and there is no plan to ensure approximately equal numbers of drinkers and nondrinkers across both conditions.
- Although the investigators provide a rationale for relying exclusively on self-reported alcohol use, this approach remains a notable weakness.

5. Environment:

Strengths

- The research environment is excellent and well suited for the proposed work.

Weaknesses

- There is no notable weakness.

Study Timeline:

Strengths

- The study timeline is reasonable and detailed with specified benchmarks and deliverables.

Weaknesses

- There is no notable weakness.

Protections for Human Subjects:

Acceptable Risks and/or Adequate Protections

Data and Safety Monitoring Plan (Applicable for Clinical Trials Only):

Acceptable

Inclusion Plans:

- Sex/Gender: Distribution justified scientifically
- Race/Ethnicity: Distribution justified scientifically
- For NIH-Defined Phase III trials, Plans for valid design and analysis: Not applicable
- Inclusion/Exclusion Based on Age: Distribution justified scientifically
    - The inclusion of females and members of diverse racial/ethnicity groups is reasonable. A justified scientific rationale is provided for the exclusion of youth < 14 years of age and adults.

Resource Sharing Plans:

Unacceptable

- Plans to submit grant-related human subjects' data to an NIAAA-sponsored data repository are not provided.

Budget and Period of Support:

Recommend as Requested

THE FOLLOWING SECTIONS WERE PREPARED BY THE SCIENTIFIC REVIEW OFFICER TO SUMMARIZE THE OUTCOME OF DISCUSSIONS OF THE REVIEW COMMITTEE, OR REVIEWERS' WRITTEN CRITIQUES, ON THE FOLLOWING ISSUES:

PROTECTION OF HUMAN SUBJECTS: ACCEPTABLE

INCLUSION OF WOMEN PLAN: ACCEPTABLE

INCLUSION OF MINORITIES PLAN: ACCEPTABLE

INCLUSION OF CHILDREN PLAN: ACCEPTABLE

COMMITTEE BUDGET RECOMMENDATIONS: The budget was recommended as requested.

RESOURCE SHARING PLANS: UNACCEPTABLE
NIAAA Resource Sharing Plan (NOT AA 18-010): NIAAA expects investigators to provide basic plans for submitting grant-related human subjects data to a NIAAA-sponsored data repository, NIAAA Data Archive ($NIAAA_{DA}$). These plans should be included in the Data Sharing Plan located in the Resource Sharing Plan section of grant applications. This applies to all grant applications (new and resubmitted) that include human subject research, except Fellowship (F), Training (T), Small Business (SBIR/STTR),

and Education (R25) grants. The data in the $NIAAA_{DA}$ will be catalogued and made available to the general research community after an embargo period.

Footnotes for 1 R01 AA027253-01A1; PI Name: Harris, Sion Kim

Ad hoc or special section application percentiled against "Total CSR" base.

NIH has modified its policy regarding the receipt of resubmissions (amended applications). See Guide Notice NOT-OD-14-074 at http://grants.nih.gov/grants/guide/notice-files/NOT-OD-14-074.html. The impact/priority score is calculated after discussion of an application by averaging the overall scores (1-9) given by all voting reviewers on the committee and multiplying by 10. The criterion scores are submitted prior to the meeting by the individual reviewers assigned to an application, and are not discussed specifically at the review meeting or calculated into the overall impact score. Some applications also receive a percentile ranking. For details on the review process, see http://grants.nih.gov/grants/peer_review_process.htm#scoring.

MEETING ROSTER

Clinical, Treatment and Health Services Research Review Subcommittee
National Institute on Alcohol Abuse and Alcoholism Initial Review Group
NATIONAL INSTITUTE ON ALCOHOL ABUSE AND ALCOHOLISM
AA-3
06/21/2019

Notice of NIH Policy to All Applicants: Meeting rosters are provided for information purposes only. Applicant investigators and institutional officials must not communicate directly with study section members about an application before or after the review. Failure to observe this policy will create a serious breach of integrity in the peer review process, and may lead to actions outlined in NOT-OD-14-073 at https://grants.nih.gov/grants/guide/notice-files/NOT-OD-14-073.html and NOT-OD-15-106 at https://grants.nih.gov/grants/guide/notice-files/NOT-OD-15-106.html, including removal of the application from immediate review.

CHAIRPERSON(S)

MIRANDA, ROBERT JR., PHD
ASSOCIATE PROFESSOR
DEPARTMENT OF PSYCHIATRY & HUMAN BEHAVIOR
CENTER FOR ALCOHOL & ADDICTION STUDIES
BROWN UNIVERSITY
PROVIDENCE, RI 02906

MEMBERS

ARIAS, ALBERT JOSEPH, MD
ASSOCIATE PROFESSOR
DEPARTMENT OF PSYCHIATRY
DIVISION OF ADDICTION PSYCHIATRY
VIRGINIA COMMONWEALTH UNIVERSITY
RICHMOND, VA 23284

BATES, MARSHA E., PHD
DISTINGUISHED PROFESSOR
DEPARTMENT OF KINESIOLOGY AND HEALTH
DIRECTOR, CARDIAC NEUROSCIENCE LABORATORY
CENTER OF ALCOHOL STUDIES
RUTGERS STATE UNIVERSITY OF NEW JERSEY
PISCATAWAY, NJ 08854-8001

BRADLEY, KATHARINE ANTHONY, MD, MPH
SENIOR INVESTIGATOR
GROUP HEALTH RESEARCH INSTITUTE
SEATTLE, WA 98101

CARROLL, KATHLEEN M., PHD
ALBERT E. KENT PROFESSOR OF PSYCHIATRY
DIRECTOR OF PSYCHOSOCIAL RESEARCH
DIVISION OF ADDICTIONS
DEPARTMENT OF PSYCHIATRY
YALE UNIVERSITY SCHOOL OF MEDICINE
WEST HAVEN, CT 06511

CHOI, DOO-SUP, PHD
PROFESSOR OF PHARMACOLOGY AND PSYCHIATRY
DEPARTMENT OF MOLECULAR PHARMACOLOGY
AND EXPERIMENTAL THERAPEUTICS
MAYO CLINIC COLLEGE OF MEDICINE
ROCHESTER, MN 55905

KELLY, JOHN F., PHD
ELIZABETH R. SPALLIN ASSOCIATE PROFESSOR OF
PSYCHIATRY IN ADDICTION MEDICINE AT HARVARD
MEDICAL SCHOOL
FOUNDER AND DIRECTOR, RECOVERY RESEARCH
INSTITUTE
ASSOCIATE DIRECTOR, CENTER FOR ADDICTION MEDICINE
MASSACHUSETTS GENERAL HOSPITAL
BOSTON, MA 02114

MURPHY, JAMES G., PHD
PROFESSOR AND DIRECTOR OF CLINICAL TRAINING
DEPARTMENT OF PSYCHOLOGY
UNIVERSITY OF MEMPHIS
MEMPHIS, TN 38152

RAY, LARA A., PHD
PROFESSOR
DEPARTMENT OF PSYCHOLOGY
UNIVERSITY OF CALIFORNIA, LOS ANGELES
LOS ANGELES, CA 90095

ZWEBEN, ALLEN, PHD
ASSOCIATE DEAN, RESEARCH AND ACADEMIC AFFAIRS
PROFESSOR OF SOCIAL WORK
SCHOOL OF SOCIAL WORK
COLUMBIA UNIVERSITY
NEW YORK, NY 10027

SCIENTIFIC REVIEW OFFICER

SRINIVAS, RANGA V., PHD
CHIEF, EXTRAMURAL PROJECT REVIEW BRANCH
EXTRAMURAL PROJECT REVIEW BRANCH
NATIONAL INSTITUTE ON ALCOHOL ABUSE AND
ALCOHOLISM
NATIONAL INSTITUTES OF HEALTH
BETHESDA, MD 20892

EXTRAMURAL SUPPORT ASSISTANT

STRINGFIELD, DONNA
EXTRAMURAL SUPPORT ASSISTANT
OFFICE OF EXTRAMURAL ACTIVITIES
NATIONAL INSTITUTE ON ALCOHOL ABUSE AND
ALCOHOLISM
NATIONAL INSTITUTES OF HEALTH
ROCKVILLE, MD 20852

Consultants are required to absent themselves from the room during the review of any application if their presence would constitute or appear to constitute a conflict of interest.
